# Author Correction: Efficient spatially targeted gene editing using a near-infrared activatable protein-conjugated nanoparticle for brain applications

**DOI:** 10.1038/s41467-023-39976-3

**Published:** 2023-07-17

**Authors:** Catarina Rebelo, Tiago Reis, Joana Guedes, Cláudia Saraiva, Artur Filipe Rodrigues, Susana Simões, Liliana Bernardino, João Peça, Sónia L. C. Pinho, Lino Ferreira

**Affiliations:** 1 CNC-Center for Neurosciences and Cell Biology, University of Coimbra, Coimbra, Portugal; 2 Faculty of Medicine, University of Coimbra, Coimbra, Portugal; 3 Health Sciences Research Centre (CICS-UBI), University of Beira Interior, Covilhã, Portugal; 4 Department of Life Sciences, University of Coimbra, Coimbra, Portugal

**Keywords:** Nanobiotechnology, Nanoparticles, Optogenetics, Gene delivery, Genetic techniques

Correction to: *Nature Communications* 10.1038/s41467-022-31791-6, published online 16 July 2022

In the Acknowledgements section, the funding source ‘BrainEdition’ should have read ‘CENTRO-01-0145-FEDER-028060 - PTDC/NAN-MAT/28060/2017 (acronym: BrainEdition)’. The original article has been corrected.

