# Supplementary material for: Efficient spatially targeted gene editing using a near-infrared activatable protein-conjugated nanoparticle for brain applications
Source: Nat Commun. 2022 Jul 16;13:4135. doi: 10.1038/s41467-022-31791-6 (PMC9287341; doi:10.1038/s41467-022-31791-6)
Supplement: Supplementary file 1 — Supplementary Information [file 41467_2022_31791_MOESM1_ESM.pdf]

Supplementary Materials for

Efficient spatially targeted gene editing using a near-infrared activatable protein-conjugated nanoparticle for brain applications

Rebelo C.[1,2], Reis T.[1], Guedes J.[1], Saraiva C.[3], Rodrigues A.F.[1], Simões, S[1], Bernardino L.[3], Peça J.[1,4], Pinho S.L.C.[1,]*, Ferreira L.[1,2,]*

Affiliations:

[1] CNC-Center for Neurosciences and Cell Biology, University of Coimbra, Portugal
[2] Faculty of Medicine, University of Coimbra, Portugal
[3] Health Sciences Research Centre (CICS-UBI), University of Beira Interior, Portugal
[4] Department of Life Sciences, University of Coimbra, Portugal
*Corresponding author: lino@uc-biotech.pt; slpinho@uc-biotech.pt

This PDF file includes:

Supplementary Figures 1-24

Supplementary Methods

Supplementary Table 1- List of viruses

Supplementary Table 2- List of antibodies

Supplementary Table 3- List of primers used for RT-qPCR

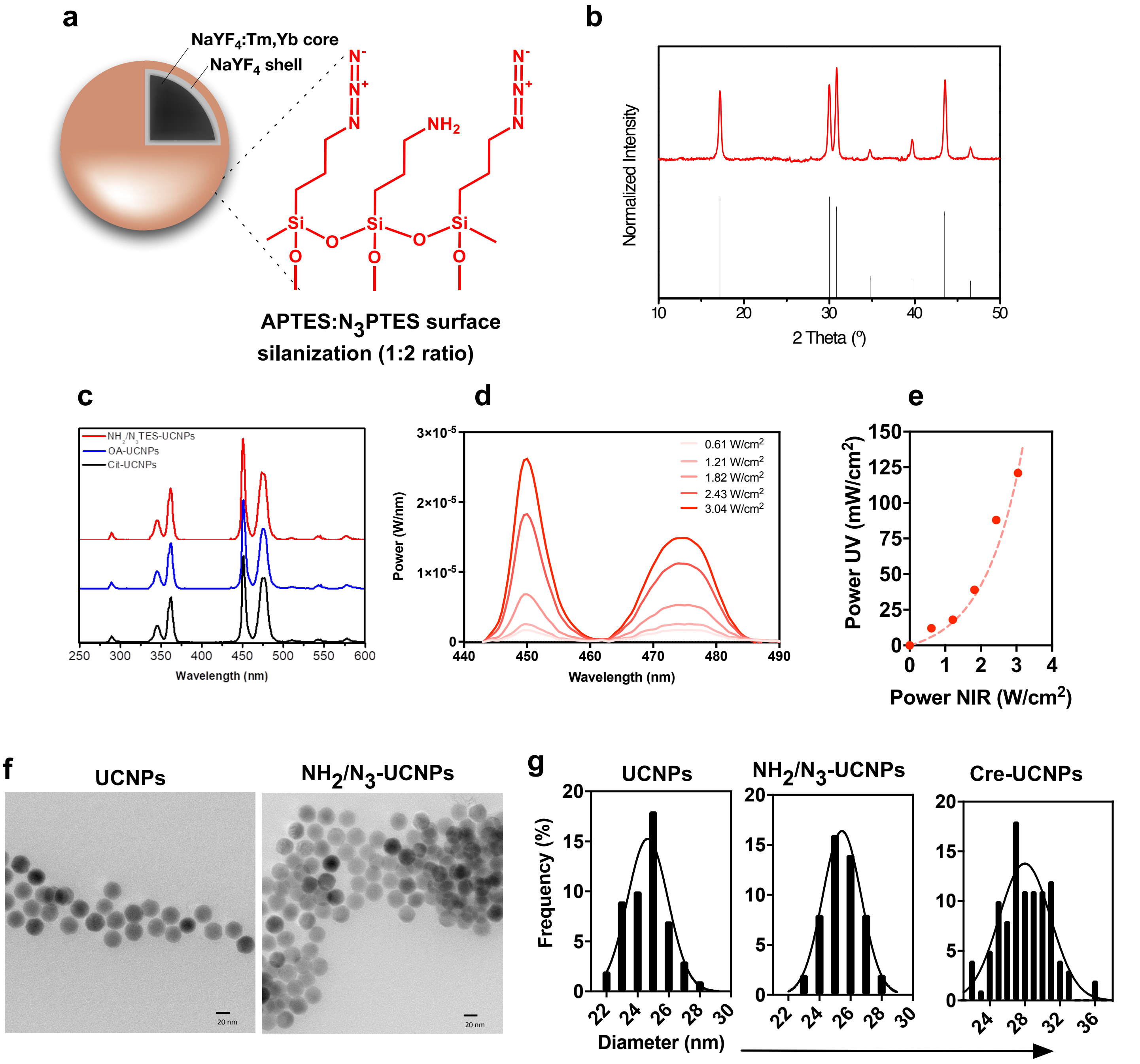

Supplementary Fig. 1 - Physicochemical characterization of UCNPs. (a) Schematic representation of UCNPs after silanization. (b) Powder X-ray diffraction (XRD) patterns for $NaYF_4$:Tm,Yb NPs (top) and β-$NaYF_4$ standard pattern (bottom). The pattern was indexed to card nº 00-011-3582. (c) Luminescence emission spectrum of oleic acid (OA)-coated UCNPs (blue), Citrate (Cit)-coated UCNPs (black) and silanized UCNPs (NH_2/N_3-UCNPs). The emission intensities were normalized in all cases. (d) Power spectral density measurement via integrating sphere. Results for different power densities of NIR-light. (e) Conversion yield for NIR to UV-light (exponential growth equation fitting; y = 14.068*exp(x/2.177) -14. (f) Representative TEM images of UCNPs and modified APTES:N_3PTES UCNPs, obtained from a total of 5 images per condition. Scale bars = 20 nm. (g) Diameter distribution of UCNPs and NH_2/N_3-UCNPs determined by TEM. The diameter distribution of Cre-UCNPs was determined by STEM. In all cases, results were obtained by measuring a total of 100 NPs from 5 TEM/STEM images.

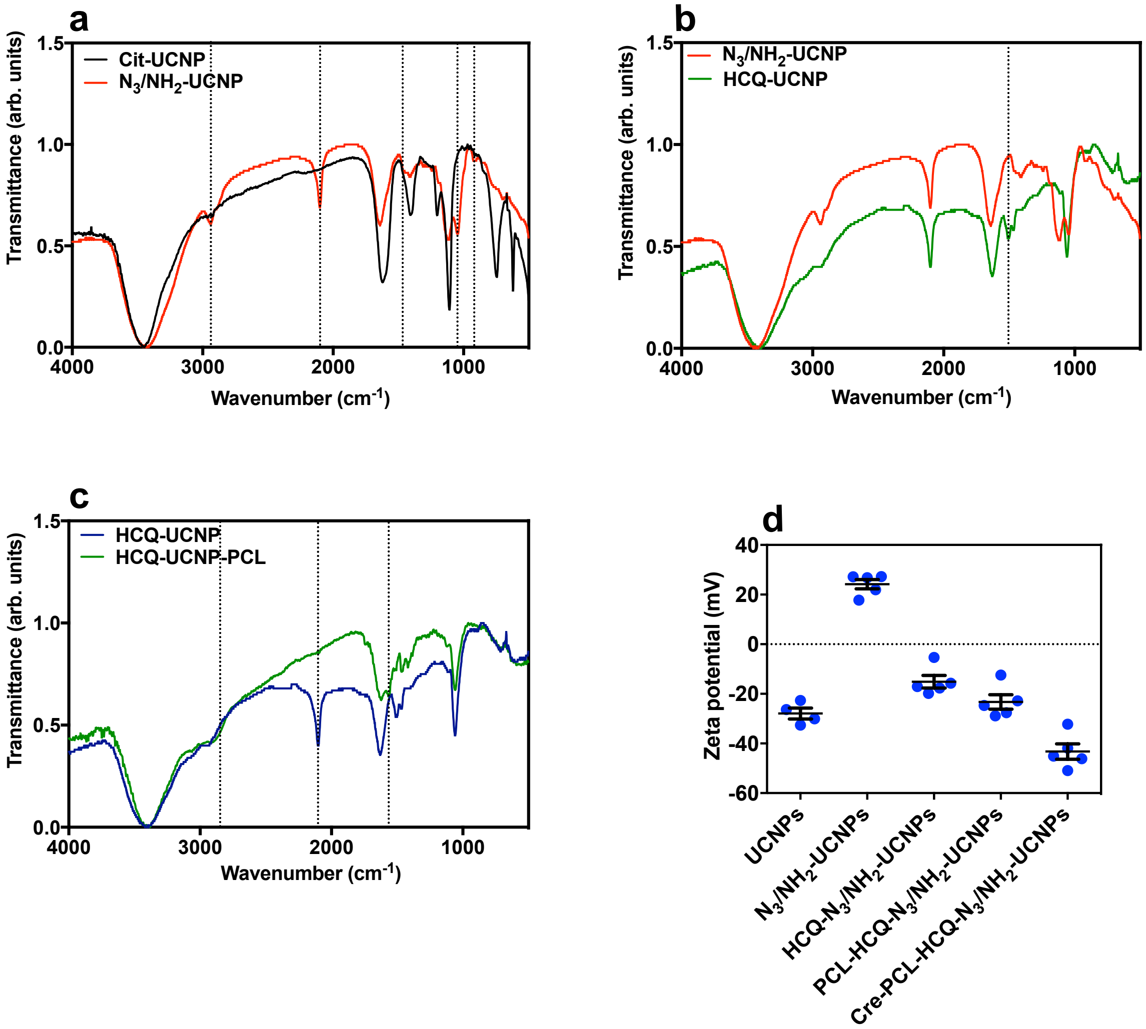

Supplementary Fig. 2 - Surface characterization of UCNPs. (a-c) FTIR analyses of the UCNPs during the modification steps: citrate (cit)-coated UCNPs (black), silane modification with N_3PTES and NH_2PTES (red), conjugation of hydroxychloroquine (green) and conjugation of photo-cleavable linker (blue). Silanization at the surface of the UCNPs could be indicated by the emergence of the bands at 2937 cm^{-1} and 2894 cm^{-1}, corresponding to the asymmetric and symmetric stretching vibrations of CH_2 groups of the silane alkyl chain, while the bands at 1042 cm^{-1} and 1110 cm^{-1} are assigned to the vibration of Si-O-Si bonds. While the presence of N_3PTES could be demonstrated by the strong band at 2105 cm^{-1} corresponding to the asymmetric stretch vibration of the azide group, the bands at 1452 cm^{-1} and 916 cm^{-1} indicating hydrogen bonding of NH_2 and N-H bending mode, respectively, ascribe to the presence of primary amines of NH_2PTES. The attachment of HCQ to UCNPs was confirmed by the appearance of the band at 1513 cm^{-1}, ascribed to C-N stretching vibrations. Finally, the conjugation of PCL could be confirmed by the reduced peak attributed to the azide group (2105 cm^{-1}), in contrast with the emergence of the bands around 2850 cm^{-1} (triazole) and 1560 cm^{-1} (N-O), indicating the successful cycloaddition of PCL with the azide groups. (d) Zeta potential analyses of UCNPs (0.5 mg/mL) in 1.0 mM KCl, pH 7. Results are average ± SEM, n=5. Silanization shifted the initially negative charge of the UCNPs (owing to citrate ions adsorbed to the NP surface) to a positive value due to the presence of amine groups from NH_2PTES. Successful conjugation of HCQ decreased the number of available amine groups, shifting the NP surface charge back to a negative value. Further conjugation of PCL and Cre recombinase did not significantly affect the overall surface charge.

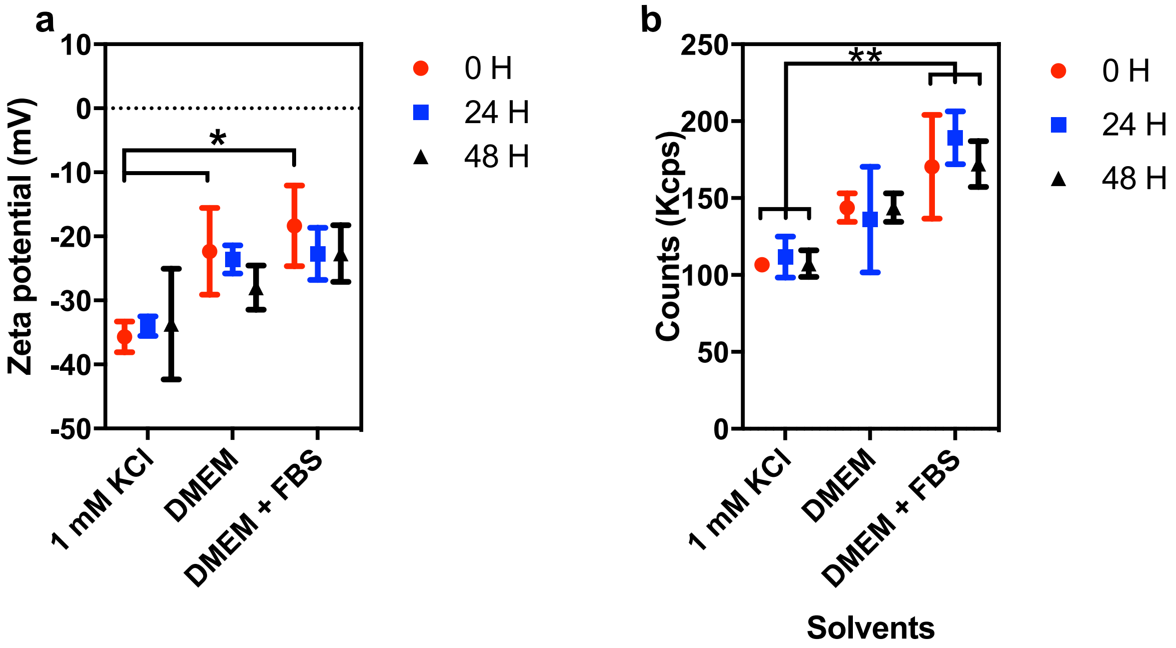

Supplementary Fig. 3 - Stability of Cre-NPs suspended in water or cell culture media. (a) Zeta potential of Cre-UCNPs suspended in water (1mM KCl), DMEM (medium used for fibroblasts), and DMEM supplemented with FBS. It should be noted that the transfection of the cells with Cre-UCNPs was always performed in serum-free media. (b) Counts (Kcps) of Cre-UCNPs suspended in water (1mM KCl), DMEM (medium used for fibroblasts), and DMEM supplemented with FBS. Counts and zeta potential determined by dynamic light scattering method (DLS) using a Zeta Plus Analyzer (Brookhaven). In a and b, results are average ± SD, n=3. Statistical analyses were performed by two-way ANOVA test followed by a Tukey´s multiple comparisons test. * and ** denotes statistical significance (*P*=0.0477 and *P*=0.0019, respectively).

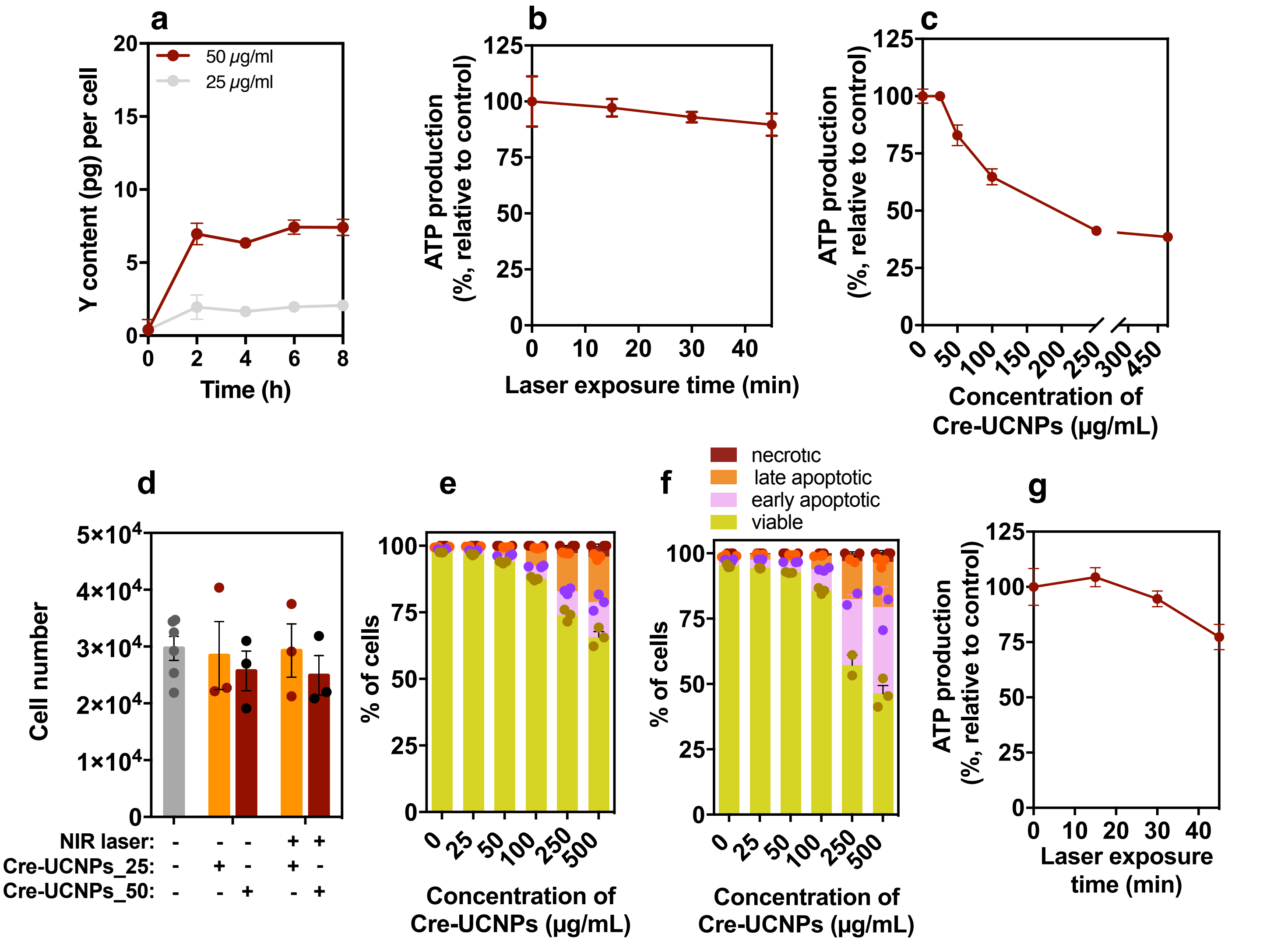

Supplementary Fig. 4 - Cytotoxicity of Cre-UCNPs and NIR laser. (a) Cellular uptake. Quantification of yttrium (Y) by ICP-MS analyses in fibroblasts exposed to different concentrations of Cre-UCNPs without HCQ. Cells were incubated with NPs (25 or 50 µg/mL) for a specific time, washed, harvested and finally freeze-dried. Concentration of yttrium was normalized per cell. Results are expressed as Mean ± SEM (n=3). (b) Cytotoxicity in fibroblasts exposed to a NIR laser for different times, as evaluated by ATP measurements at 48 h after laser exposure. Results are Mean ± SEM (n=3). Irradiation for up to 45 min did not significantly affect ATP production in irradiated cells.(c) Cytotoxicity of several concentrations of Cre-UCNPs (non-irradiated; with HCQ) in fibroblasts, as evaluated by ATP measurements. Fibroblasts were incubated for 4 h, washed, and ATP production was evaluated after 48 h. Results are Mean ± SEM (n=3-4 independent experiments). Cre-UCNPs were tolerated at concentrations ≤ 50 µg/mL, with minimal impact on cell metabolism. (d) Quantification of fibroblast number after incubation with Cre-UCNPs (25 or 50 *µ*g/mL) for 4 h, washed, activated or not with a NIR laser, and cell number was evaluated at time 24 h. Results are expressed as Mean ± SEM (n = 3). No statistical significance was found after performing a two-way ANOVA test followed by Bonferroni's multiple comparisons test, suggesting that cell number was not affected after treatment with Cre-UCNPs at concentrations ≤ 50 µg/mL. (e) Cytotoxicity of UCNPs in fibroblasts evaluated by Annexin V/PI analyses. The percentage of apoptotic and necrotic cells was determined by flow cytometry. Fibroblasts were incubated with Cre-UCNPs (e and f) for 4 h, washed to remove the non-internalized UCNPs and finally allowed to grow for 24 h (e) or 48 h (f). Percentages of live (annexin V−/PI−), early apoptotic (annexin V+/PI−), late apoptotic (annexin V+/PI+) and necrotic (annexin V−/PI+) cells were obtained. Results are expressed as Mean ± SEM (n = 3). Our results indicate that Cre-UCNPs at 25 *µ*g/mL (even at 50 *µ*g/mL) had no significant effect in cell apoptosis and necrosis. (g) Cytotoxicity of irradiated Cre-UCNPs in fibroblasts, as evaluated by ATP measurements. Fibroblasts were incubated with Cre-UCNPs (25 *µ*g/mL) for 4 h, washed, irradiated for a specific time, and ATP production was evaluated after 48 h. Results are Mean ± SEM (n=3). No significant alterations were observed after irradiation for 15-30 min) with a NIR-laser (980 nm, 785 mW/cm^{-2}).

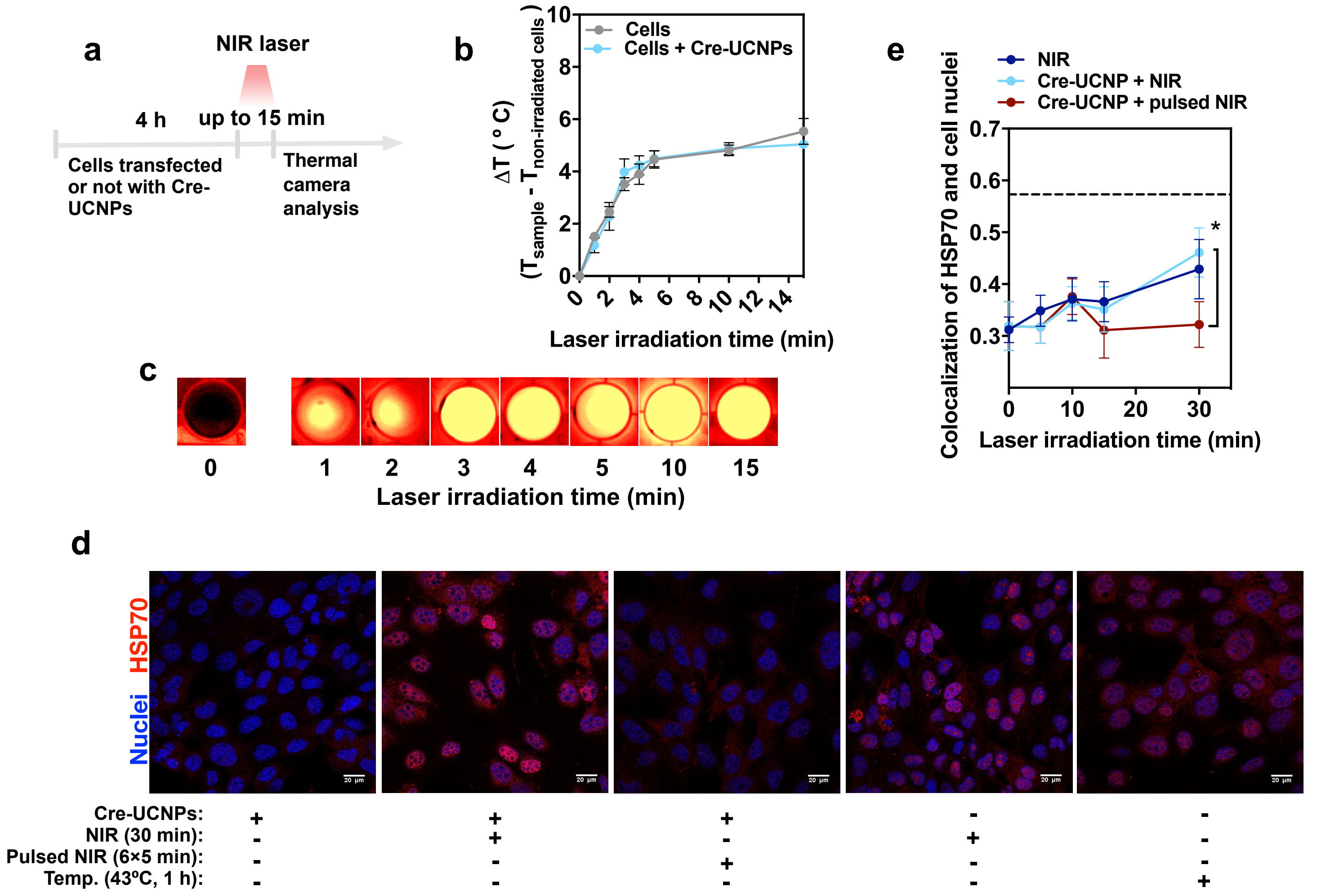

Supplementary Fig. 5 - Cytotoxicity of NIR radiation and Cre-UCNPs. (a) Schematic representation of the experimental set up to monitor cell temperature after NIR exposure. Fibroblasts were incubated for 4 h with Cre-UCNPs (50 µg/mL), washed to remove the non-internalised NPs, and then exposed to a NIR laser (980 nm, 785 mW/cm^2; until 15 min of continuous irradiation). After irradiation, media was removed from the cells and they were analysed under a thermal camera. As a control, cells not exposed to Cre-UCNPs were irradiated for the same time (1, 2, 3, 4, 5, 10 and 15 min). (b) Temperature variation in cells alone or cells transfected with Cre-UCNPs after NIR laser irradiation (980 nm, 785 mW/cm^2) for different times. Results are expressed as Mean ± SEM (n = 3-5 wells per time and condition from 1 independent experiment). Our results indicate that the temperature of the cells increased approximately 5°C during 15 min of irradiation, irrespective of the treatment with UCNPs. (c) Representative thermal camera images of cells incubated with Cre-UCNPs before and after NIR laser irradiation. (d and e) Colocalization of HSP70 with cell nuclei. Fibroblasts were incubated for 4 h with Cre-UCNPs (50 μg/mL), washed to remove the non-internalised NPs, and then exposed or not to a NIR laser (980 nm, 785 mW/cm^2; pulsed or continuous). Representative immunocytochemistry images of HSP70 expression in the different experimental groups, obtained from 3 independent experiments. Cells cultured in normal conditions present a diffuse pattern of HSP70 in their cytoplasm and in the nucleus. High colocalization between HSP70 and cell nuclei indicates a heat-stress response. Dashed line represents the average colocalization result for a heat shock of 43ºC of 1 h. Scale bar = 20 µm. In (e), results are Mean ± SEM (n=3-6 wells per time and condition, 2 images per well). Statistical analyses were performed by one-way ANOVA followed by a Tukey´s multiple comparisons test. * denotes statistical significance (P=0.0484). No heat-shock stress was observed in cells after exposure to NIR laser (with or without Cre-UCNPs) for times ≤ 15 min. Our results suggest that heat-shock stress response can be prevented by applying a pulsed NIR activation (6 × 5 min, 5 min interval per pulse).

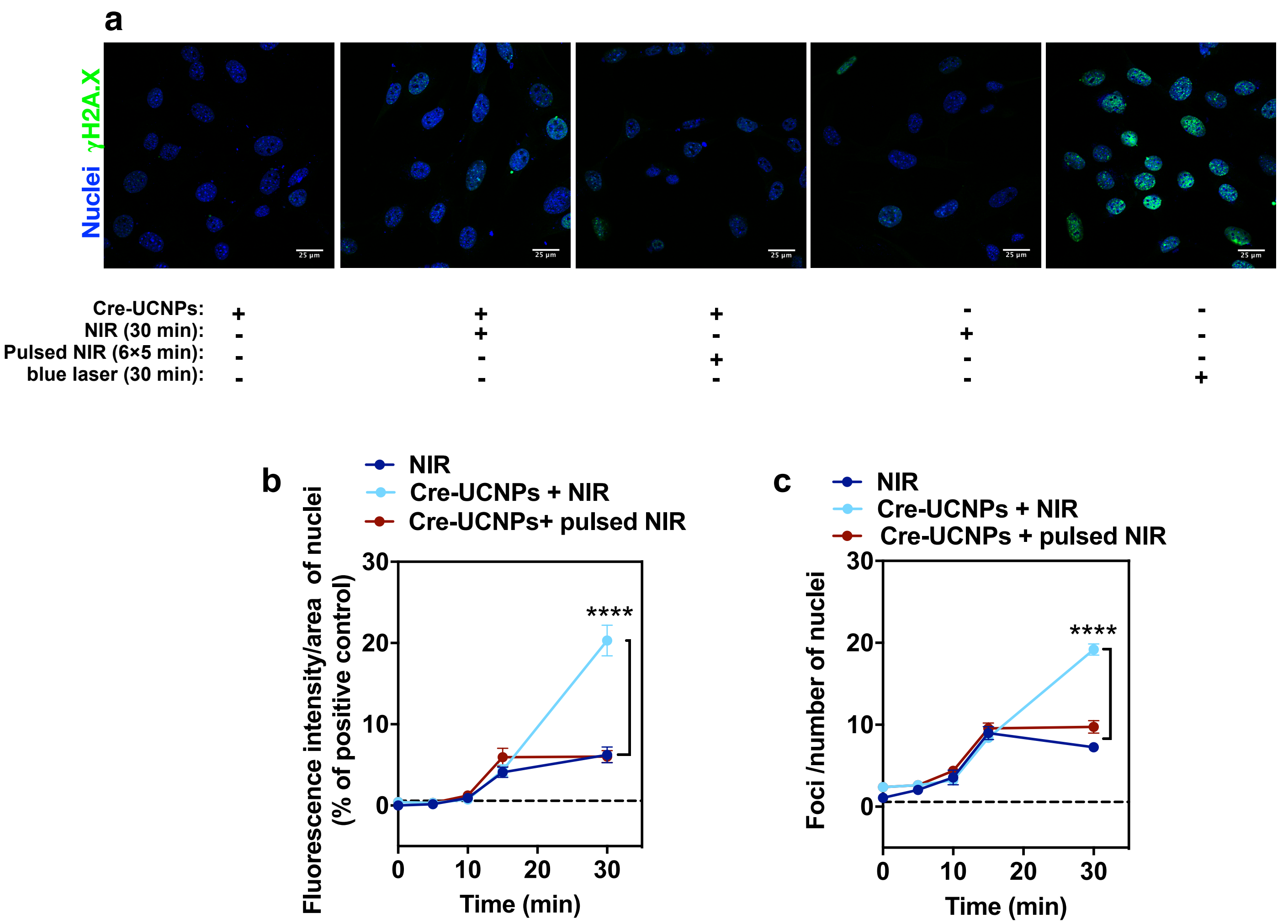

Supplementary Fig. 6 - DNA damage caused by NIR radiation and Cre-UCNPs. Cre-UCNPs emit locally UV/blue light which may induce double-stranded DNA breaks (DSBs). Therefore we quantified DSBs by assessing the phosphorylation of the histone H2A.X variant (Ser-139 residue). For this purpose, fibroblasts were incubated for 4 h with Cre-UCNPs (50 *µ*g/mL), washed to remove the non-internalised NPs, and then exposed or not to a NIR laser (980 nm, 785 mW/cm^{2}; pulsed or continuous until 30 min of irradiation). As positive control, cells irradiated with a 405 nm blue laser for 30 min. As negative control, cells not transfected with Cre-UCNPs were irradiated with a NIR laser for a specific time (i.e, 5, 10, 15 and 30 min). (a) Representative immunofluorescence images for γH2A.X on fibroblasts either transfected or not with Cre-UCNPs exposed to NIR laser for 30 min, obtained from a total of 12 images per condition. Scale bars = 25 µm. (b) γH2A.X fluorescence intensity was normalized by the area of cell nuclei and expressed as a percentage of the positive control (i.e., cells irradiated with a 405 nm blue laser for 30 min). (c) Total number of γH2A.X foci (calculated by Image J, using the Find Maxima tool; noise tolerance: 50) normalized by the number of cell nuclei. In b and c, results are Mean ± SEM (n = 3 wells per condition, 4 microscopy images per well, each image had 425,000 *µ*m^{2}). Statistical analyses were performed by One-way ANOVA test followed by a Tukey´s multiple comparisons test. **** denotes statistical significance at P<0.0001. Our results indicate that UV light emitted by Cre-UCNPs cannot induce double-stranded DNA breaks on cells if they are exposed to NIR radiation (980 nm, 785 mW/cm^{2}) for less than 30 min. The DNA damage effect observed when irradiation time is above or equal to 30 min could be reduced in case of a pulsed NIR laser activation of Cre-UCNPs within cells (6x5 min).

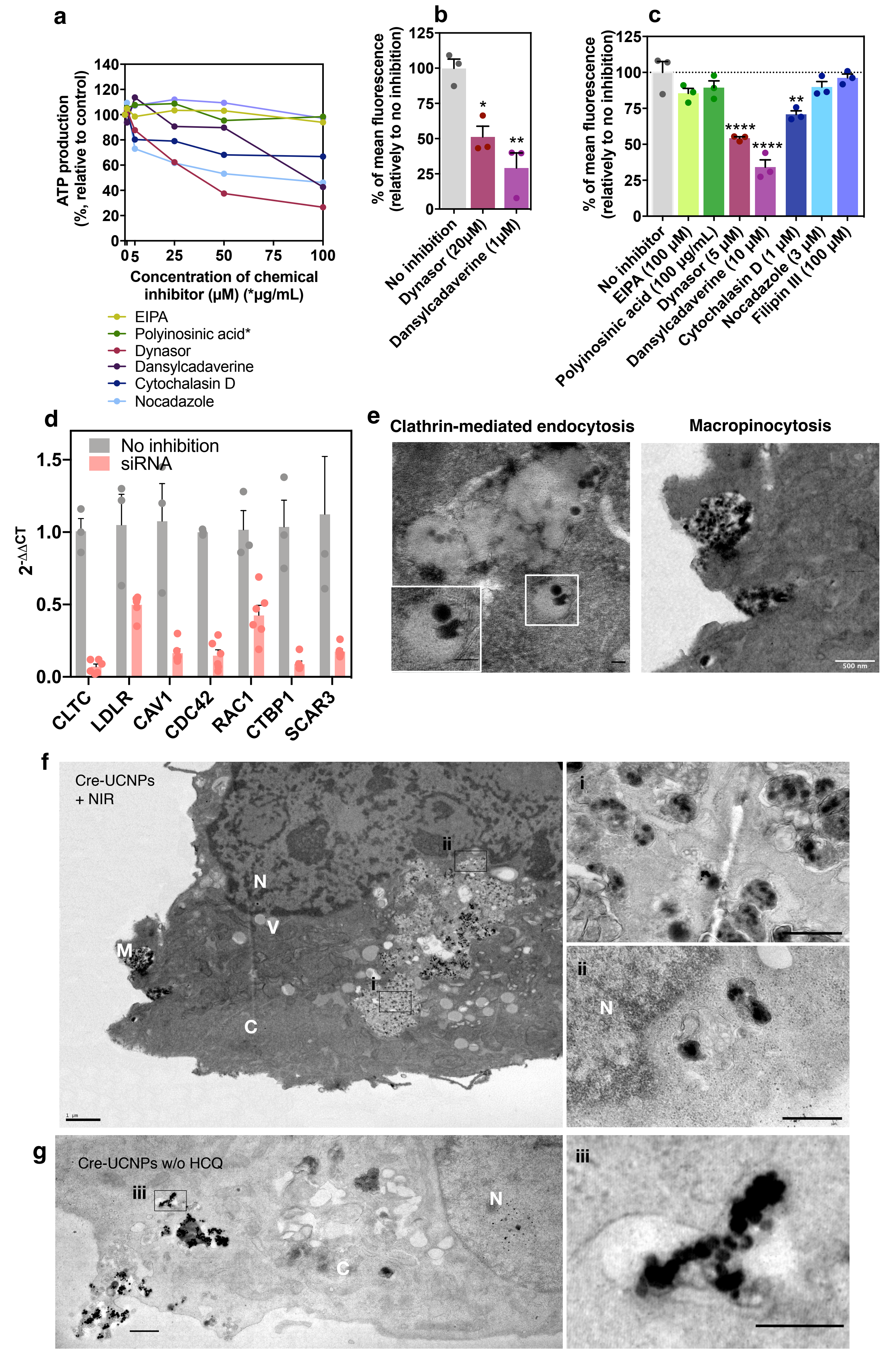

Supplementary Fig. 7 - Cellular uptake and trafficking of Cre-UCNPs. (a) Cytotoxicity of chemical inhibitors against fibroblasts. Cells were cultured in medium supplemented with different concentrations of chemical inhibitors for 24 h. Cell viability was evaluated by an ATP production kit. Results are presented as the average of 2 independent runs. (b) Transport of FITC-labeled transferrin known to selectively enter cells via clathrin-mediated endocytosis. Results are expressed as Mean ± SEM (n = 3). Statistical analysis was performed by one-way ANOVA followed by a Dunnett's multiple comparisons test, with *, ** denoting statistical significance (P=0.0117, P=0.0019, respectively). Dynasor and dansylcadaverine at concentration of 20 µM and 1 µM, respectively inhibited the internalization of transferrin (1 µg/mL) in fibroblasts. Cells were exposed to culture medium with or without dynasor/dansylcadavarine for 30 min, exposed to transferrin for 8 min, and finally characterized by flow cytometry. (c) Uptake of Cre-UCNPs (25 µg/mL) by fibroblasts in the presence of several endocytosis inhibitors. Cells were incubated with fluorescent-labeled Cre-UCNPs for 4 h in the presence of endocytosis chemical inhibitors, at non-cytotoxic concentrations, and then characterized the cells by flow cytometry. Results are Mean ± SEM (n = 3). Statistical analysis was performed by one-way ANOVA followed by a Dunnett's multiple comparisons test, with **, **** denoting statistical significance (P=0.0011, P<0.0001, respectively). Dynasor and dansylcadaverine treatment (clathrin-mediated endocytosis inhibitors) reduced the uptake of Cre-UCNPs by 46% and 66%, respectively, while cytochalasin-D (F-actin inhibitor) reduced 30% as compared to control cells. (d) qRT-PCR analyses showing the siRNA-mediated knockdown efficiency (24 h post-transfection with siRNA). Results are expressed as Mean ± SEM (n = 3). (e) Representative TEM images indicating the cellular uptake of Cre-UCNPs, obtained from a total of 18 images. NPs were found in vesicles of ~100 nm in diameter while others were being taken up by macropinocytosis. Black scale bar is 50 nm and white scale bar is 500 nm. Representative TEM images indicating the intracellular location of Cre-UCNPs (f) with and (g) without HCQ in fibroblasts, obtained from a total of 18 images. Cells were incubated with the NPs for 4 h. Scale bars = 1 µm (200 nm for insets i, ii, and iii). N: cell nucleus, C: cytoplasm, M: macropinocytosis, V: vacuole.

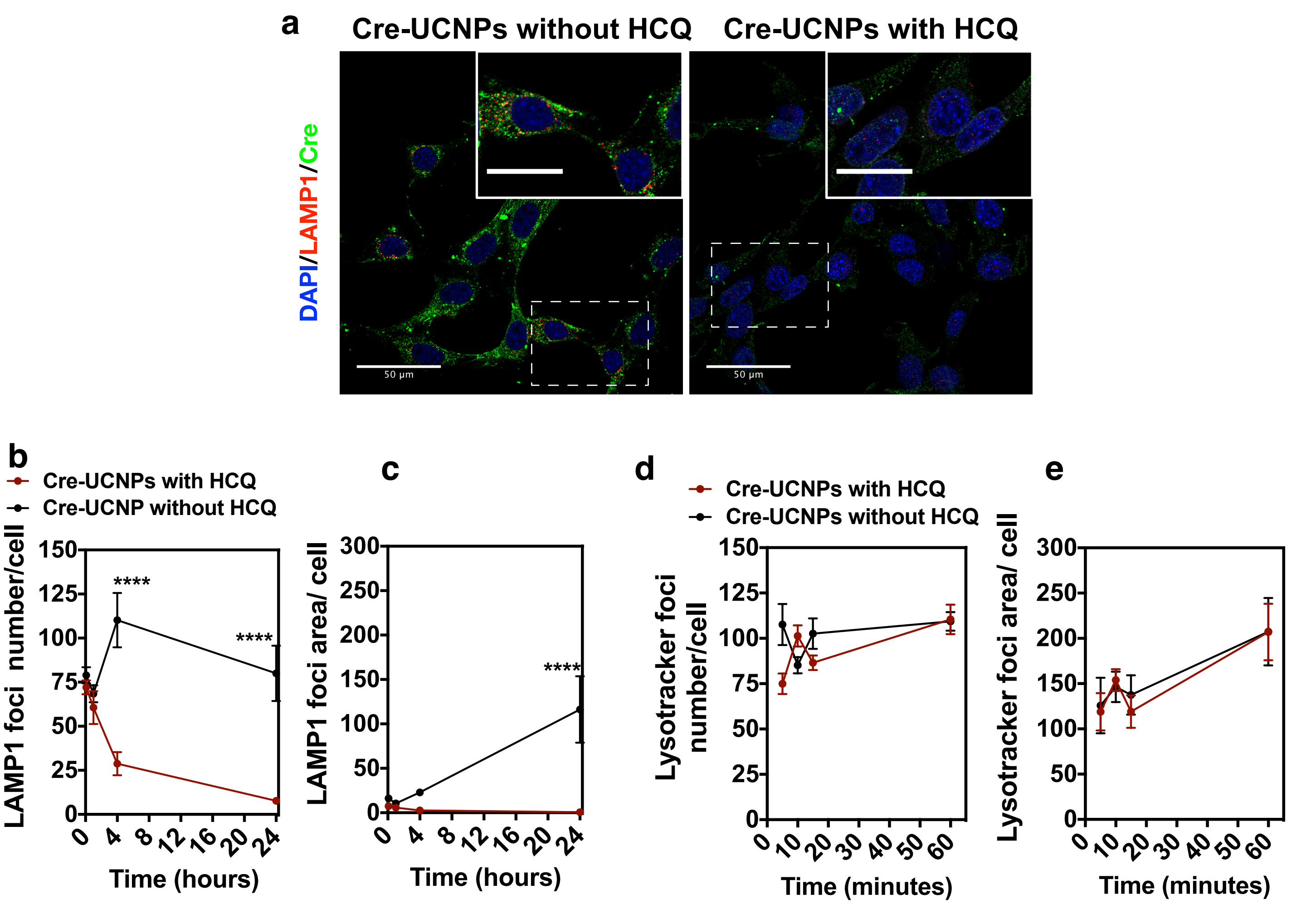

Supplementary Fig. 8 - Intracellular trafficking of Cre-UCNPs with and without HCQ. Fibroblasts were incubated with Cre-UCNPs with or without immobilised HCQ (50 µg/mL) for different time points and then washed extensively, fixed, stained with a LAMP1 antibody, and characterised by confocal microscopy. For time 24 h in b.1 and b.2, cells were exposed for 4 h to Cre-UCNPs / Cre-HCQ-UCNPs, washed, cultured for additional 20 h and then processed has mentioned above. (a) Representative fluorescence images for LAMP1 staining in cells exposed for 4 h to Cre-UCNP with HCQ or Cre-UCNP without HCQ, obtained from a total of 18 images per condition. Scale bars = 50 µm (inset = 25 µm). (b) Number (b) and area (c) of LAMP1 foci per cell in cells exposed to Cre-UCNPs with or without HCQ. Results are expressed as Mean ± SEM (n = 3 wells, for each well 6 photos with an area of 41333 µm^2 were analyzed). **** denote statistical significance (P<0.0001) as assessed by one-way ANOVA followed by a Tukey´s multiple comparisons post-test. Number (d) and area (e) of Lysotracker positive foci per cell in cells exposed to Cre-UCNPs with or without HCQ. In this experiment, fibroblasts were stained with Lysotracker Red (50 nM) for 30 min. After the staining, cells were incubated with Cre-UCNPs with or without immobilized HCQ (50 µg/mL) for different time points and then washed extensively, fixed, and characterised by fluorescence microscopy. Results are Mean ± SEM (n = 3 wells, for each well 6 photos with an area of 41333 µm^2 were analyzed). No statistical significance was found after performing one-way ANOVA followed by a Tukey´s multiple comparisons post-test. Our results indicate that the incorporation of HCQ in Cre-UCNPs did not affect affect lysosomal pH, maintaining the number of Lysotracker (a pH-sensitive endolysosomal dye) puncta in fibroblasts over a period of up to 1 h after treatment.

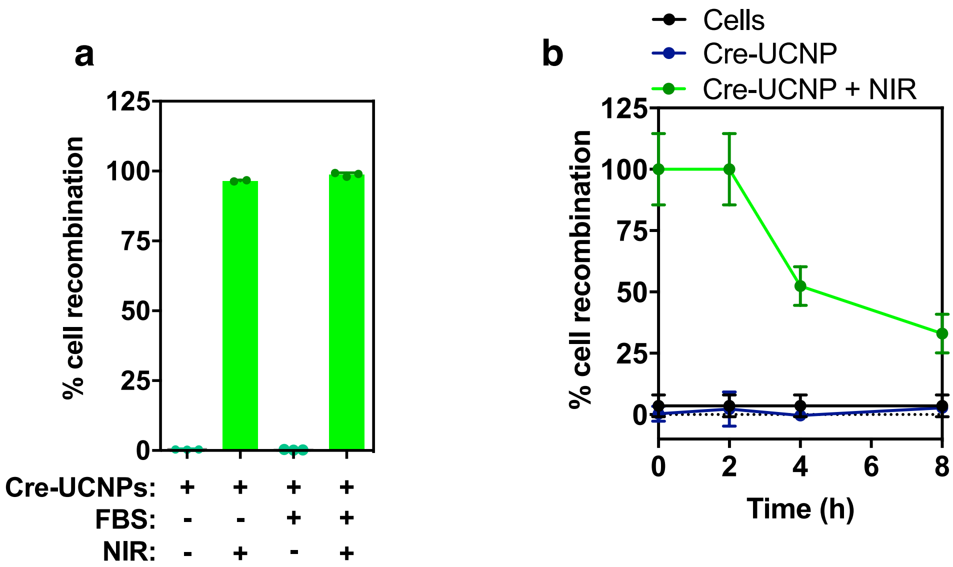

Supplementary Fig. 9 - Effect of serum and time of intracellular release of Cre within cells. (a) Quantification of cell recombination after transfection of Cre-UCNPs in the presence of serum. Fibroblast reporter cells were transfected with Cre-UCNPs (50 µg/mL) for 4 h in medium supplemented with and without fetal bovine serum (FBS; 10%, v/v), washed, activated by a NIR laser (980 nm; 785 mW/cm^2; 15 min). GFP cell fluorescence was quantified by high-content microscopy at time 48 h. Results are Mean ± SEM (n=3 independent experiments). (b) Quantification of cell recombination after NIR activation over time, following transfection of Cre-UCNPs (50 µg/mL) for 4 h in medium without FBS. Cell recombination was evaluated by flow cytometry. Results are Mean ± SEM (n=3 independent experiments).

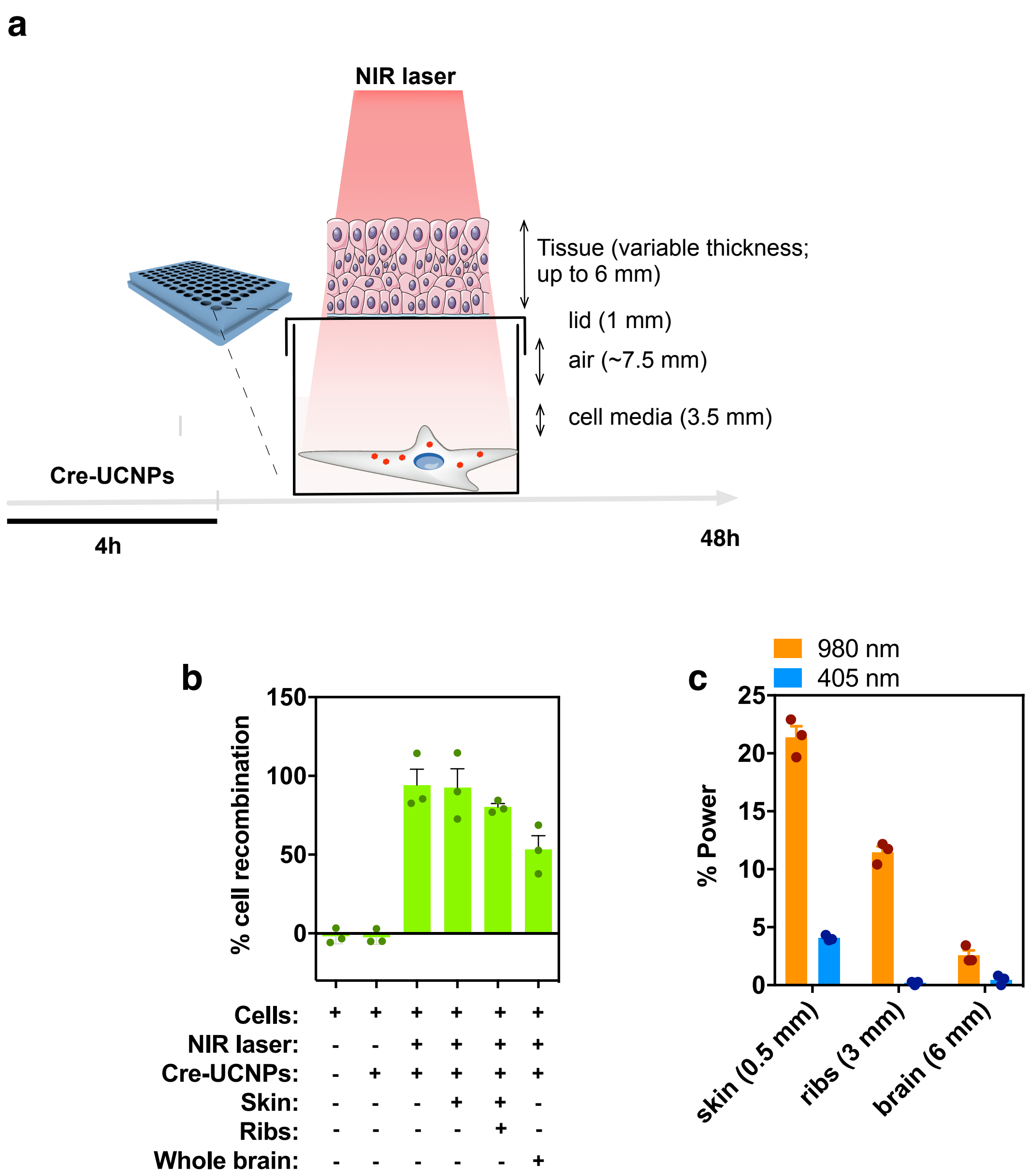

Supplementary Fig. 10 - NIR laser (980 nm) attenuation through murine tissue. (a) Schematic representation of the experimental setup. C57BL/6J mice were euthanised followed by hair shaving and removal of dorsal skin and ribs, as well as the brain. Both tissues were fixed in 4% PFA and kept in cold PBS until further analyses. The thickness of the skin, ribs and whole brain was 0.5, 3 and 6 mm, respectively, as measured by a caliper. In the day of the experiment, the tissues were placed on top of the 96-well plate lid containing the Cre reporter fibroblasts transfected with Cre-UCNPs (50 µg/mL) for 4 h. The cells with the tissue barrier were then irradiated with a 980 nm laser at 785 mW/cm^2 for 3 cycles of 5 min. (b) Percentage of fibroblast cell recombination as measured by a high-content microscope though the evaluation of the intensity of GFP (at 48 h). Results are expressed as Mean ± SEM (n = 3 wells per each condition). (c) Percentage of power density after crossing skin (0.5 mm), ribs (3 mm) and the whole mice brain (6 mm). For the laser attenuation studies, each tissue was placed in a plastic petri dish on top of a thermal power sensor (Thorlabs, s310c). Both tissues were then irradiated with a 405 nm or a 980 nm lasers during 1 min. Laser attenuation values were calculated by normalising against laser power values obtained with the empty petri dish. The thickness of the tissues was measured by a caliper. Results are expressed as Mean ± SEM (n = 3 measurements per condition).

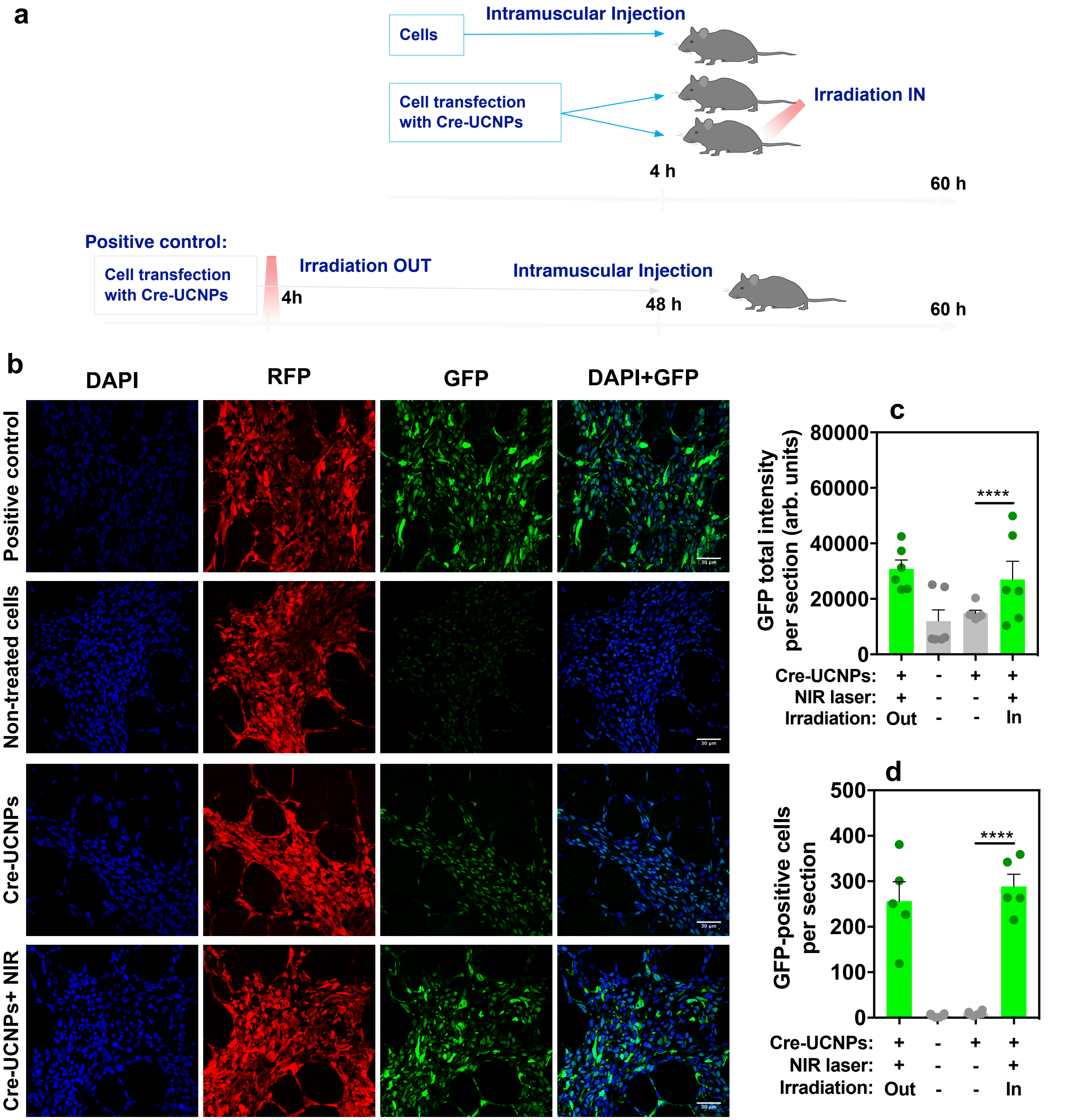

Supplementary Fig. 11 - *In vivo* activation of Cre-UCNPs. (a) Fibroblast LoxP-RFP-Stop-LoxP-GFP reporter cells were transfected with Cre-UCNPs (50 µg/mL) for 4 h, washed to remove the non-internalised Cre-UCNPs, trypsinized and finally transplanted (total of 1.5 x10^6 cells per animal; 25 μL) in the muscle of C57BL/6 mice (approximately 4 mm deep from the skin). Immediately after transplantation, the site of injection was activated or not by a NIR laser at 980 nm (425 mW/cm^2; 3 cycles of 5 min irradiation) directly above the injection site and then allowed to recombine for 60 h. As a positive control, reporter cells were activated by a NIR laser *in vitro* and allowed to express the reporter for 48 h before transplantation. Cells without transfection with Cre-UCNPs were used as negative controls. (b) Representative immunofluorescence images of mouse muscle tissue transplanted with fibroblast reporter cells 60 h post-transplantation, obtained from a total of 36 images per condition. Scale bars = 30 µm. Total GFP intensity (c) and number of GFP-positive cells (d) in each section. Results are Mean ± SEM (n=5-6 animals, with 2 sections analyzed per animal and 3 images acquired in each section; the area of each photo was 45176 μm^2). **** denotes a statistical difference (P<0.0001) by one-way ANOVA test followed by a Bonferroni´s multiple comparisons test.

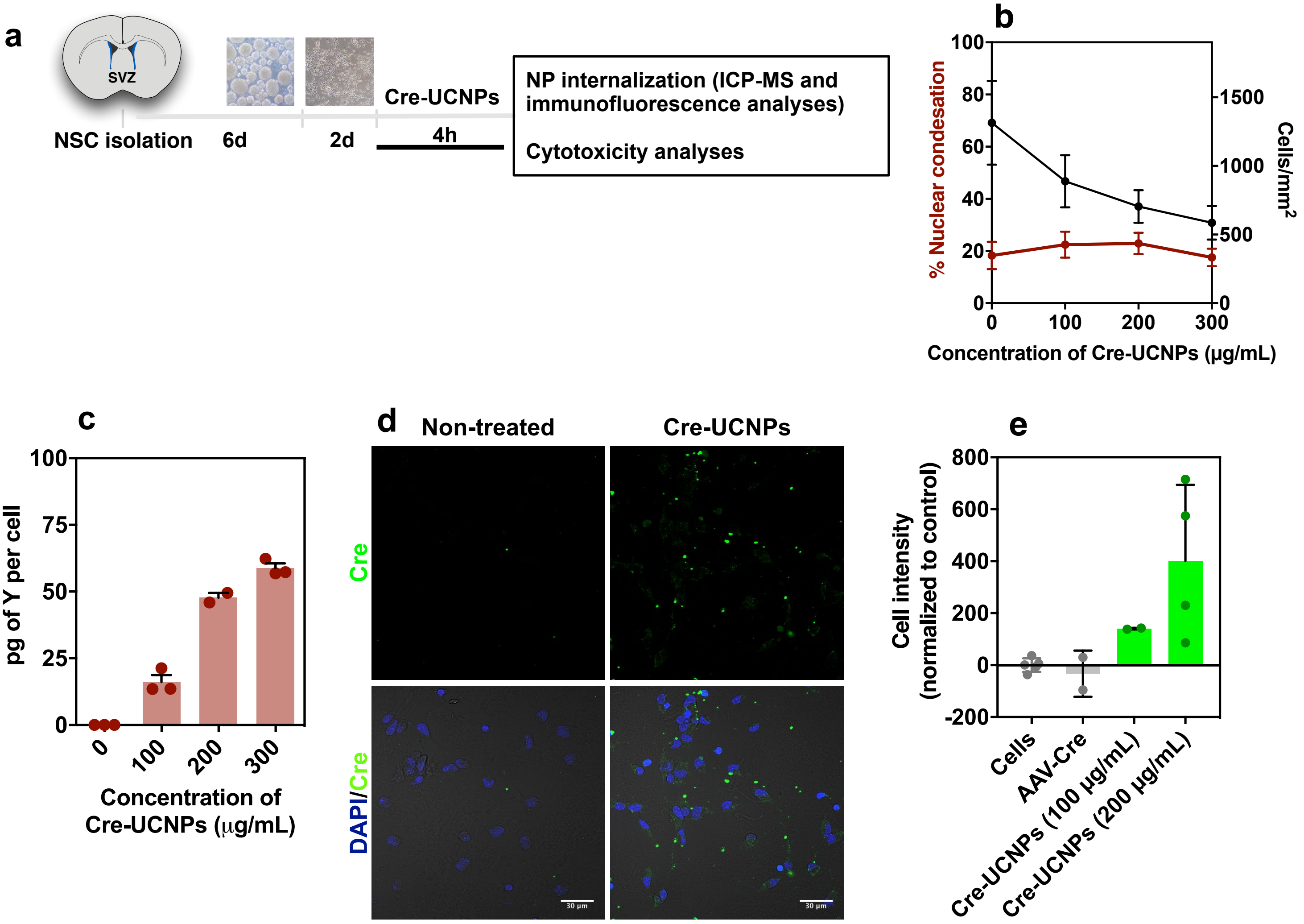

Supplementary Fig. 12 - Gene edition in SVZ primary cell cultures by Cre-UCNPs. (a) Schematic representation of the experimental setup. SVZ neurospheres were isolated from R26YFP mice, cultured in suspension for 6 days, dissociated and cultured in a dish (as adherent cells) for 2 additional days. Cells were then transfected with Cre-UCNPs for 4 h, washed to remove the non-internalised NPs and characterised by ICP-MS and immunofluorescence analyses. For cytotoxicity analyses, after NP washing, the cells were cultured for additional 4 days and then observed by microscopy for cell number and nuclear condensation (DAPI staining). (b) Cytotoxicity of Cre-UCNPs in SVZ cells was evaluated by quantifying cell number and nuclear condensation (with DAPI staining). Results are Mean ± SEM (n=3). Our results indicate that although Cre-UCNPs did not significantly affect viability of SVZ cells (assessed by nuclear condensation), cell proliferation was affected at higher concentrations (up to 300 μg/mL) after 4 days post NP exposure. (c) Cre-UCNPs uptake was determined by quantification of yttrium (Y) by ICP-MS analyses. Concentration of yttrium was normalized per cell. Results are expressed as Mean ± SEM (n=3). (d) Representative immunofluorescence images of Cre recombinase within cells, obtained from a total of 4 images per condition. Scale bars = 30 μm. (e) Quantification of Cre recombinase concentration within cells. Cre recombinase fluorescence intensity in cell cytoplasm was determined in cells transfected with different concentrations of Cre-UCNPs. As negative controls, cells without Cre-UCNPs were used. As positive controls, viral particles (AAV5.CMV.PI.Cre.rBG) encoding Cre recombinase were used. Results were normalised to negative control and expressed as Mean ± SEM (n=2-4).

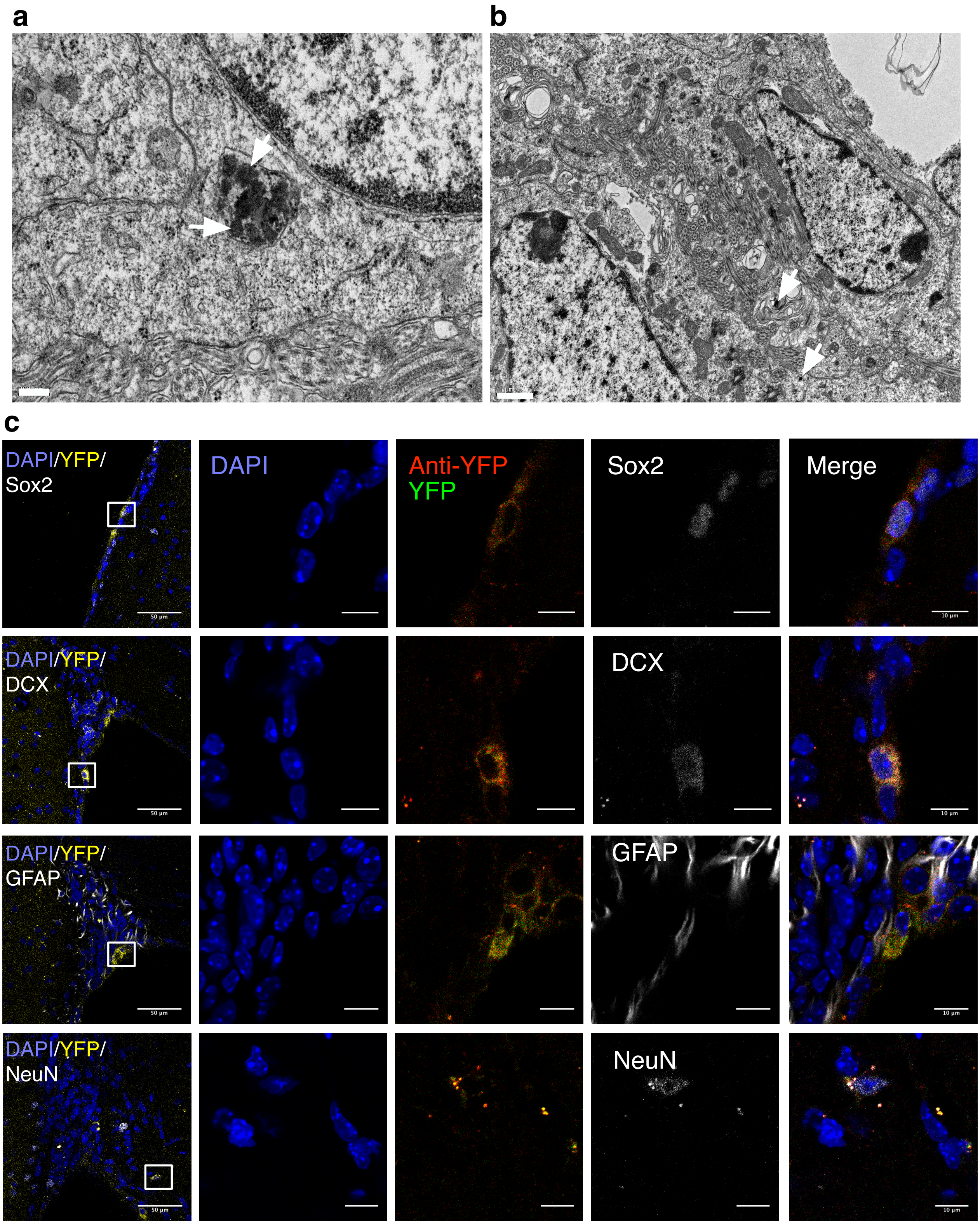

Supplementary Fig. 13 - *In vivo* activation of Cre-UCNPs following SVZ administration. (a) Representative TEM analyses showing the intracellular accumulation of Cre-UCNPs in SVZ cells, obtained from a total of 12 images. Scale bars = 200 nm and 1 μm in (a) and (b), respectively. Arrows indicate the presence of Cre-UCNPs (their composition was validated by EDX analyses). (c) Representative confocal microscopy images of mouse brain sections from SVZ region after administration of Cre-UCNPs, obtained from a total of 16 images. Our results indicate that different SVZ cell populations were edited by Cre-UCNPs (yellow-fluorescent cells). The SVZ cell populations identified by immunostaining against Sox2 (stem cell like population), doublecortin (DCX, immature neurons), GFAP (astrocyte marker) and NeuN (mature neurons). Scale bars = 50 μm (first column) and 10 μm (remaining images).

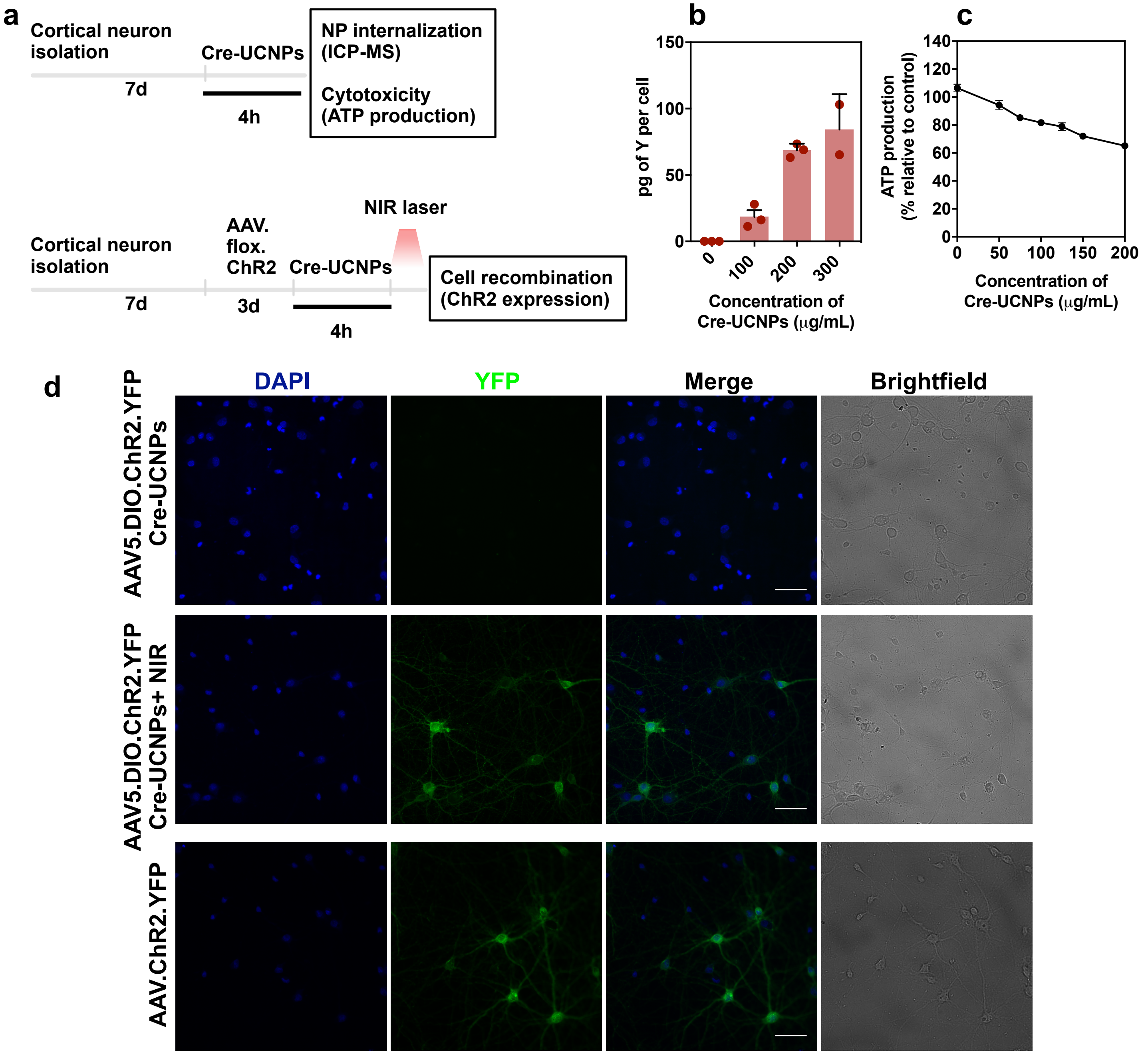

Supplementary Fig. 14 - Gene edition in cortical neuronal cell cultures by Cre-UCNPs. (a) Schematic representation of the experimental setup. Cortical neurons were isolated from brain cortices of Wistar rat embryos (E17-E19) and cultured for 7 days before tests. For internalization analyses, after 7 days, the cells were transfected with Cre-UCNPs for 4h, washed to remove the non-internalised NPs and characterized by ICP-MS. For cytotoxicity assessment, neurons were cultured for additional 2 days and analyzed for ATP production. Cre-UCNPs ability to induce gene edition was tested on neurons previously infected with AAV particles for the expression of ChR2 dependent on Cre recombinase. For this purpose, neurons were infected with AAV5.DIO.ChR2.YFP (1.48x10^10 GC/mL) for 3 days before Cre-UCNPs transfection and NIR laser exposure (785 mW/cm^2; 15 min). (b) Cre-UCNPs uptake, was determined by quantification of yttrium (Y) by ICP-MS analyses. Concentration of yttrium was normalized per cell. Results are expressed as Mean ± SEM (n=2-3). (c) Cytotoxicity analyses of Cre-UCNPs. Cells were exposed to different concentrations of Cre-UCNPs for 4 h, washed to remove the non-internalised NPs and cultured for another 44 h. At the end, cell viability was measured using the CellTiter-Glo ATP kit according to the manufacturer's instructions. Results are Mean ± SEM (n=3). (d) Representative fluorescence images of YFP expression after recombination in cortical neurons, obtained from a total of 15 images. Recombination was observed to be dependent on NIR laser photoactivation. As a positive control neurons were infected with AAV9.ChR2-YFP for direct expression of ChR2 (i.e., independent of Cre recombinase). Scale bars = 50 μm.

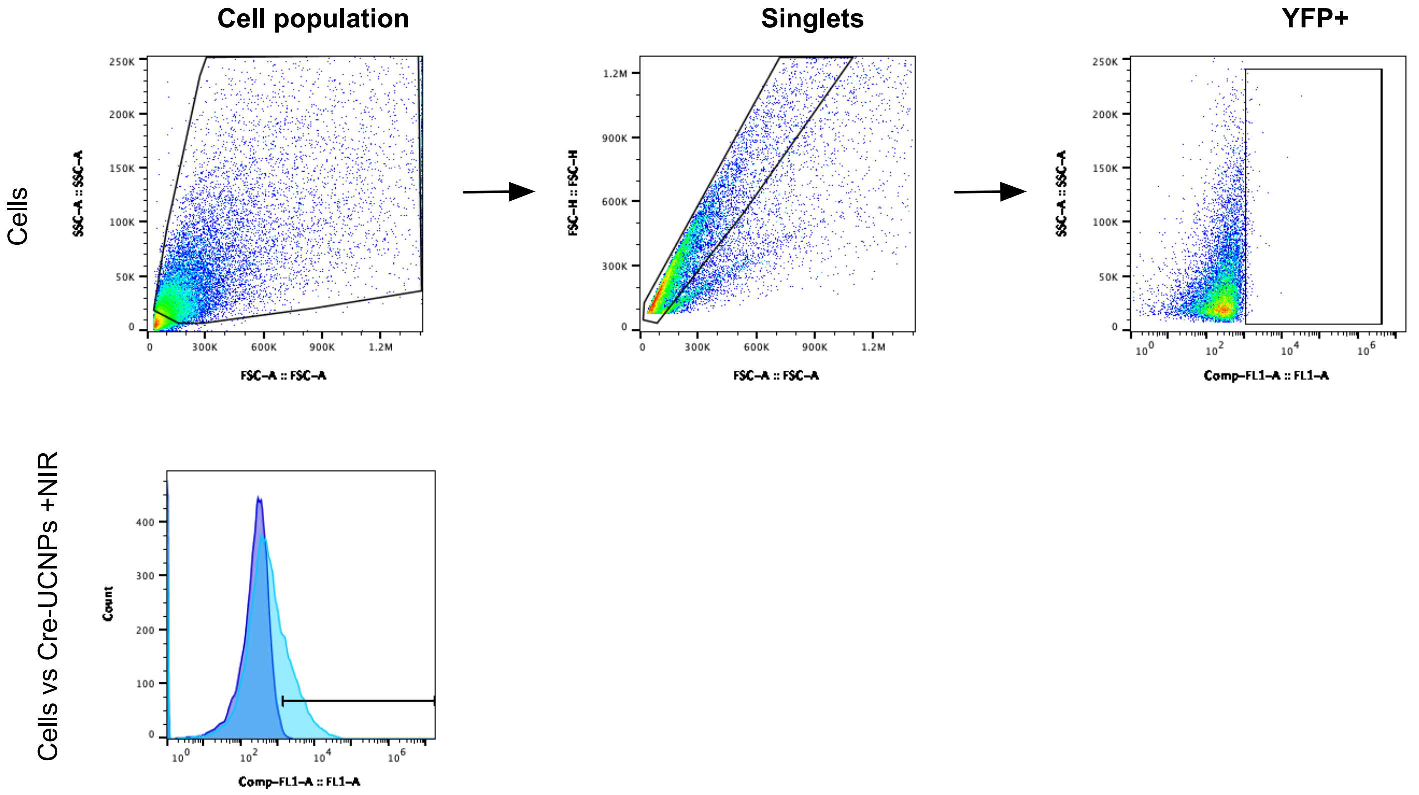

Supplementary Fig. 15 - Gating strategy for the definition of YFP^{+} cells in Fig. 5b.

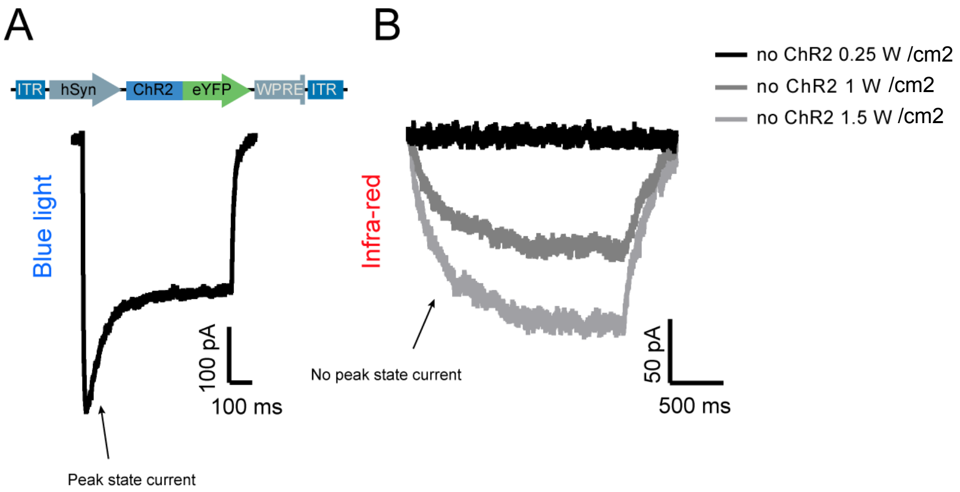

Supplementary Fig. 16 - Light-mediated currents measurements. A cortical neuronal culture was used to validate blue light-dependent ChR2 depolarizations currents. After isolation cells were cultured for 7 days before treatments. AAV5.ChR2-YFP was added to the medium and let for ChR2-YFP expression for 7 days. (a) Voltage-clamp currents generated from blue-light stimulus. The current shows a peak current, characteristic of ChR2 depolarization. (b) Voltage-clamp currents generated from NIR-light stimulus. A cortical neuronal culture without ChR2-YFP expression was used to validate NIR light-dependent depolarization currents. The current doesn't show the characteristic peak of ChR2 depolarizations.

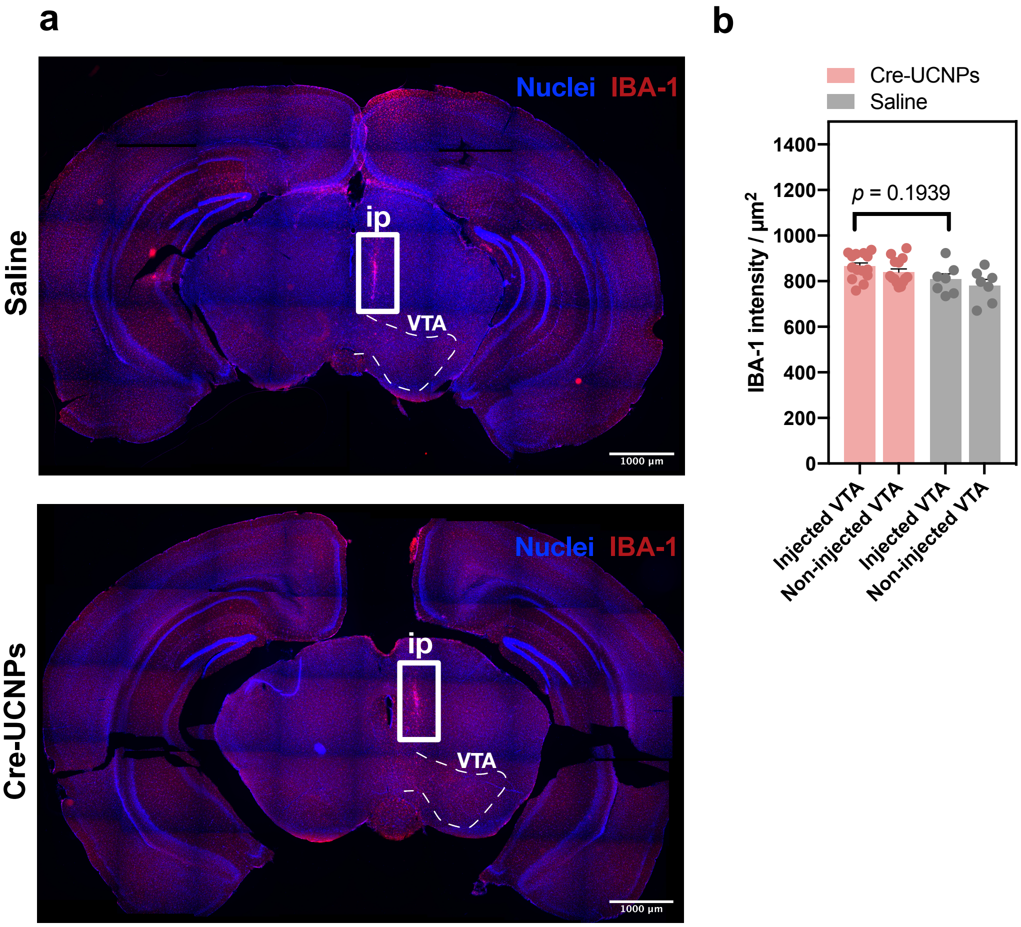

Supplementary Fig. 17 - Microglia activation in response to Cre-UCNPs following administration in the VTA. C57BL/6 mice were subjected to stereotaxic surgery in the VTA. Animals were divided into two groups: the Cre-UCNPs+NIR group received the nanoparticles (30 µg) and NIR irradiation, while the sham group received only a saline solution injection in the same coordinates. Staining of IBA-1 (microglial marker) was performed 3 days after injections. (a) Representative images of IBA-1 staining, obtained from a total of 12 images per condition, showing the "injection path" ROI (ip) with high microglia activation and the VTA ROI defined for the analyses. Injection ROI was defined as a 200 µm square region surrounding "injection path" microglia activations. Scale bars = 1000 μm. (b) IBA-1 intensity was determined by fluorescence microscopy, and normalized to the ROI area. IBA-1 intensity was observed in the VTA targeted region. Results are expressed as Mean ± SEM (n=4 animals for Cre-UCNPs and 3 animals for Saline group, with 4 brain slices analyzed per animal). No statistical difference was observed after performing one-way ANOVA test followed by a Tukey´s multiple comparisons test.

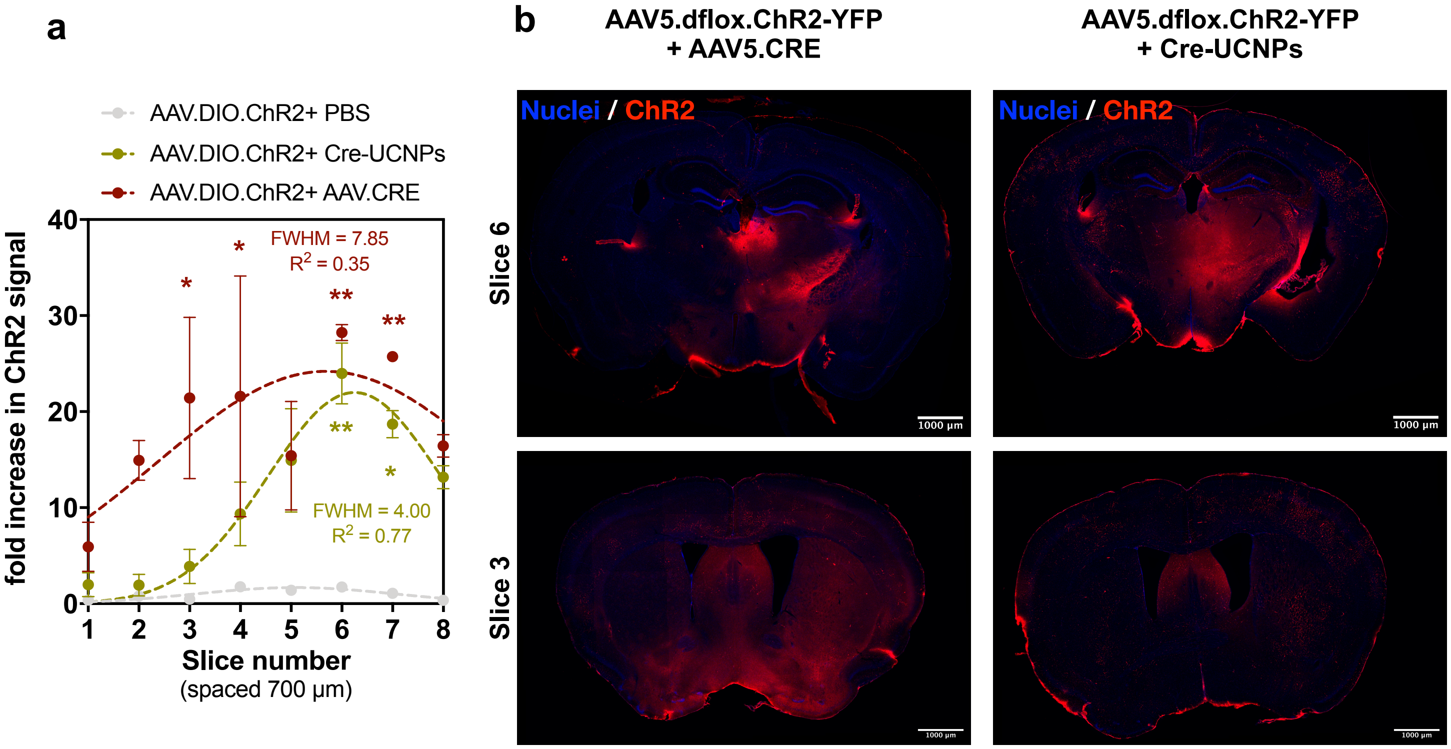

Supplementary Fig. 18 - Expression of ChR2 in other regions of the brain after Cre-UCNPs administration in the VTA. C57BL/6 mice were subjected to stereotaxic surgery in the VTA. Animals were divided into three groups: the AAV.DIO.ChR2+Cre-UCNPs group received the conditional virus for the expression of ChR2 together with the nanoparticles (30 µg) and NIR irradiation, while the control groups received a second virus for the expression of Cre or no second virus. The injections were performed sequentially in the same coordinates of the VTA (corresponding to slice number 6-7). Tissues were collected 30 days after injections and stained for ChR2 to evaluate the expression of the channel in non-target areas. 8 brain slices spaced by 700 µm were chosen for each animal. (a) ChR2 signals were quantified in images of whole brain slices and fitted to a Gaussian distribution, evidencing a more localised expression of ChR2 around the injection site after Cre-UCNP activation (FWHM = 4.00 slices, corresponding to approximately 2.8 mm) compared to AAV.CRE (FWHM = 7.85), with a marked decrease in other regions away from the target area. This could be attributed to the broader tropism of AAV, whereas Cre-mediated recombination from UCNPs is spatially targeted by NIR activation. Results are expressed as Mean ± SEM (n=3 animals for Cre-UCNPs and AAV.CRE; n=1 animal for PBS negative control). (*) and (**) denote statistical significance compared to the corresponding negative control at each slice (P=0.0373 and P=0.0084, respectively), as assessed by one-way ANOVA followed by a Tukey´s multiple comparisons test. (b) Representative images of ChR2 expression near the injection site (slice 6) and away from the injection (slice 3), obtained from 3 independent experiments. Scale bars = 1000 µm. These images illustrate the local expression of ChR2 induced by Cre-UCNP activation around the injection site, in slices 6 (p = 0.0084 vs PBS) and 7 (p = 0.0373), whereas AAV.CRE induced significant ChR2 expression in more distant regions, as shown in slices 3 (p = 0.0190) and 4 (p = 0.0266).

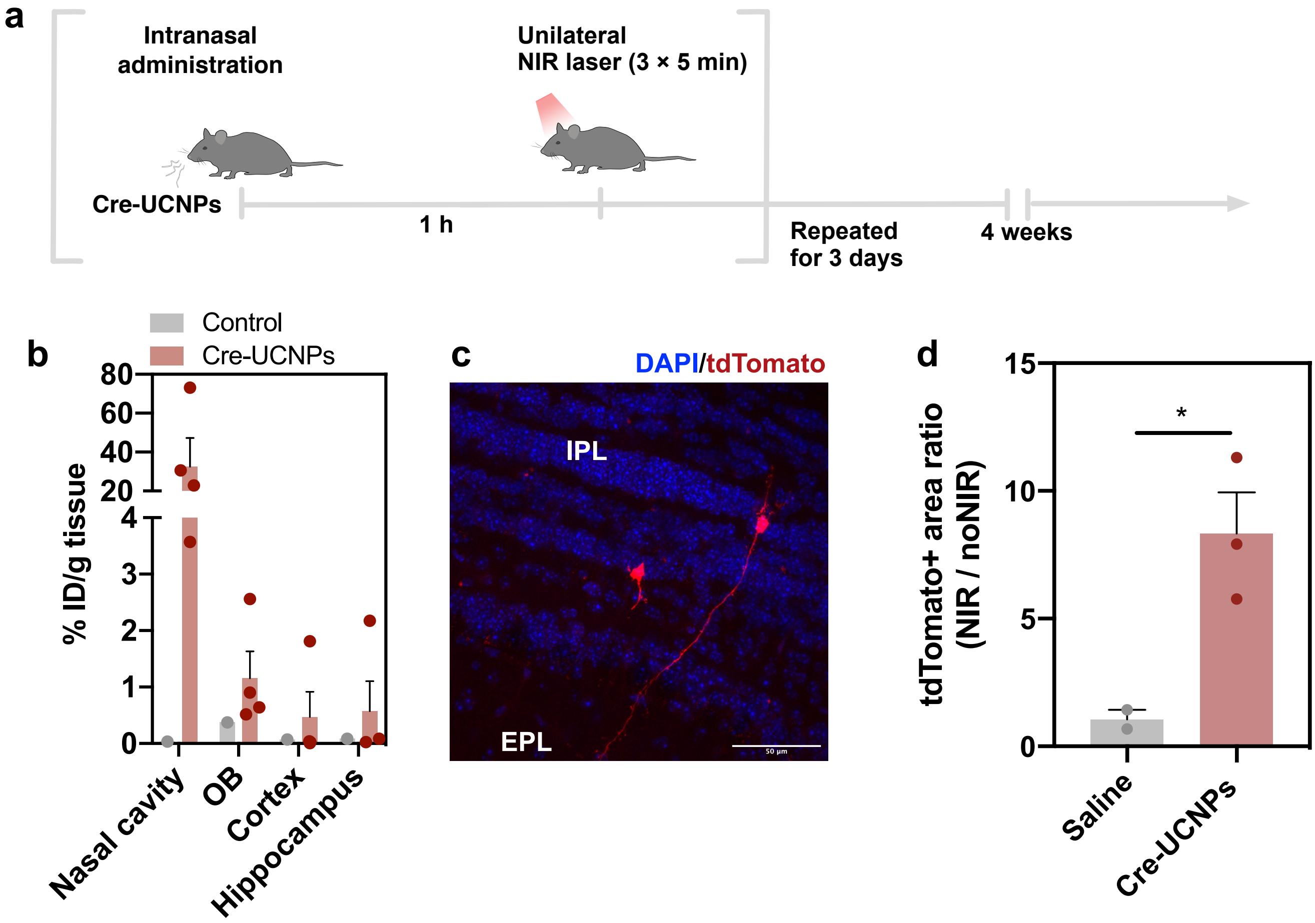

Supplementary Fig. 19 - Intranasal administration of Cre-UCNPs and NIR-mediated recombination. (a) R26tdTomato mice were subjected to intranasal administrations of the Cre-UCNPs over the time of three days. Animals were divided into two groups: the Cre-UCNPs+NIR group received the nanoparticles (120 µg), while the control group received only a saline solution. Each day, animals were subjected to the NIR laser exposure (3 x 5 min) 1 h after administration. (b) Quantification of Cre-UCNPs in the brain after intranasal administration. C57BL/6 mice were subjected to a single intranasal instillation of Cre-UCNPs and analysed by ICP-MS. Animals were divided into two groups: (i) Cre-UCNPs+NIR group (120 µg) and control group (saline). Yttrium content was determined by ICP-MS in the nasal cavity, olfactory bulb (OB), cortex and hippocampus, 4 h after administration. Results are expressed as % ID (initial dose of Y) per gram of tissue. Mean ± SEM (n=4 animals for Cre-UCNPs and 1 animal for control group). (c) Representative image of Cre-mediated recombination following NIR activation in only 1 side of the OB, obtained from 3 independent experiments. Recombination resulted in the expression of tdTomato, whose fluorescence was evaluated 4 weeks after administration. Scale bars = 50 *µ*m. EPL: external plexiform layer, IPL: internal plexiform layer. (d) Quantification of tdTomato fluorescent areas in the NIR-exposed side was normalised to the unexposed side of the OB (noNIR), in order to validate the spatial resolution of NIR-mediated recombination. Results are expressed as Mean ± SEM (n= 3 animals treated with Cre-UCNPs and 2 with Saline; 6 images were obtained from each animal). Statistical analysis was performed by a two-tailed unpaired t test, with * denoting statistical significance (P = 0.0406).

a

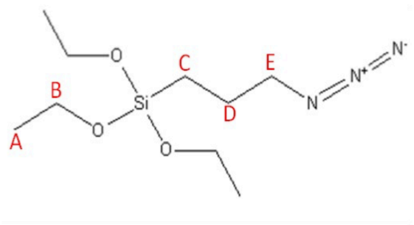

^{1}H NMR (400 MHz, Chloroform-*d*) δ 3.79 (q, *J* = 6.9 Hz, 6H), 3.24 (t, *J* = 6.4 Hz, 2H), 1.74 – 1.60 (m, 2H), 1.26 – 1.12 (m, 9H), 0.64 (dt, *J* = 11.7, 6.3 Hz, 2H).

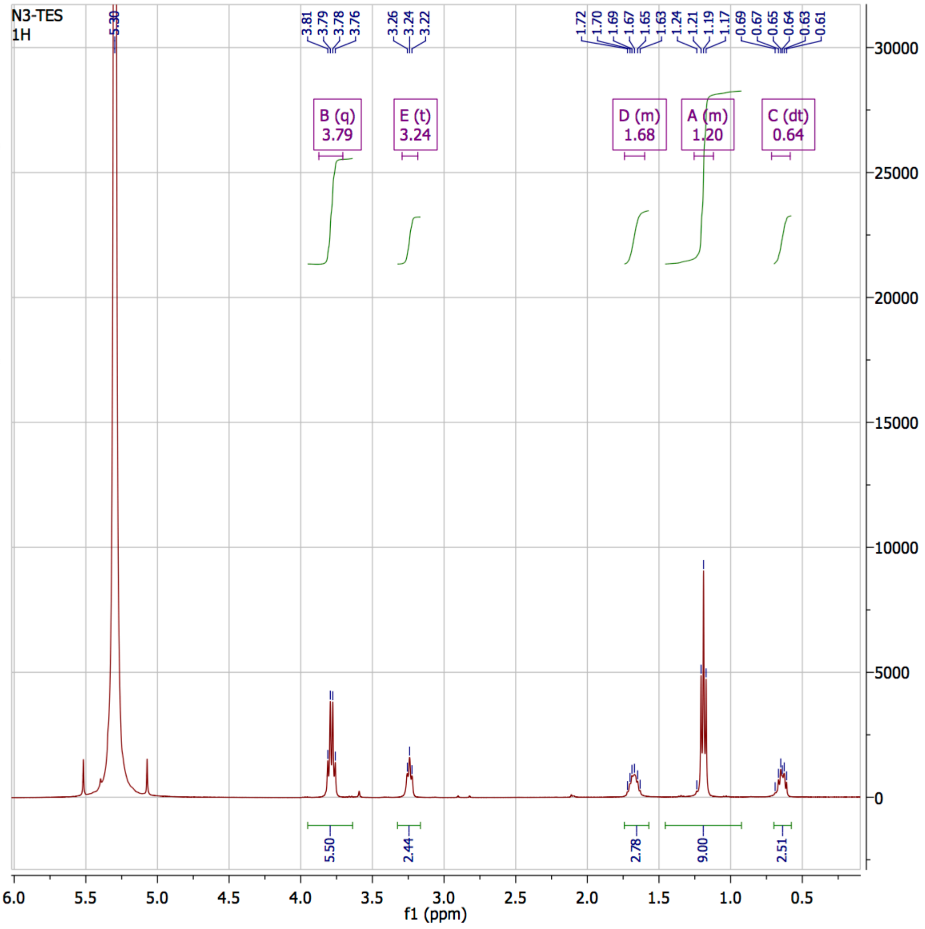

b

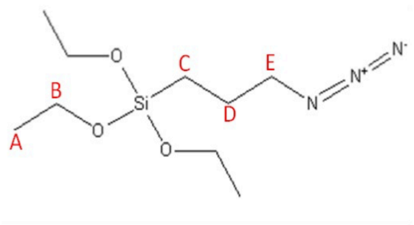

^{13}C NMR (101 MHz, $CDCl_3$) δ 58.15, 53.76, 22.61, 18.26, 7.54

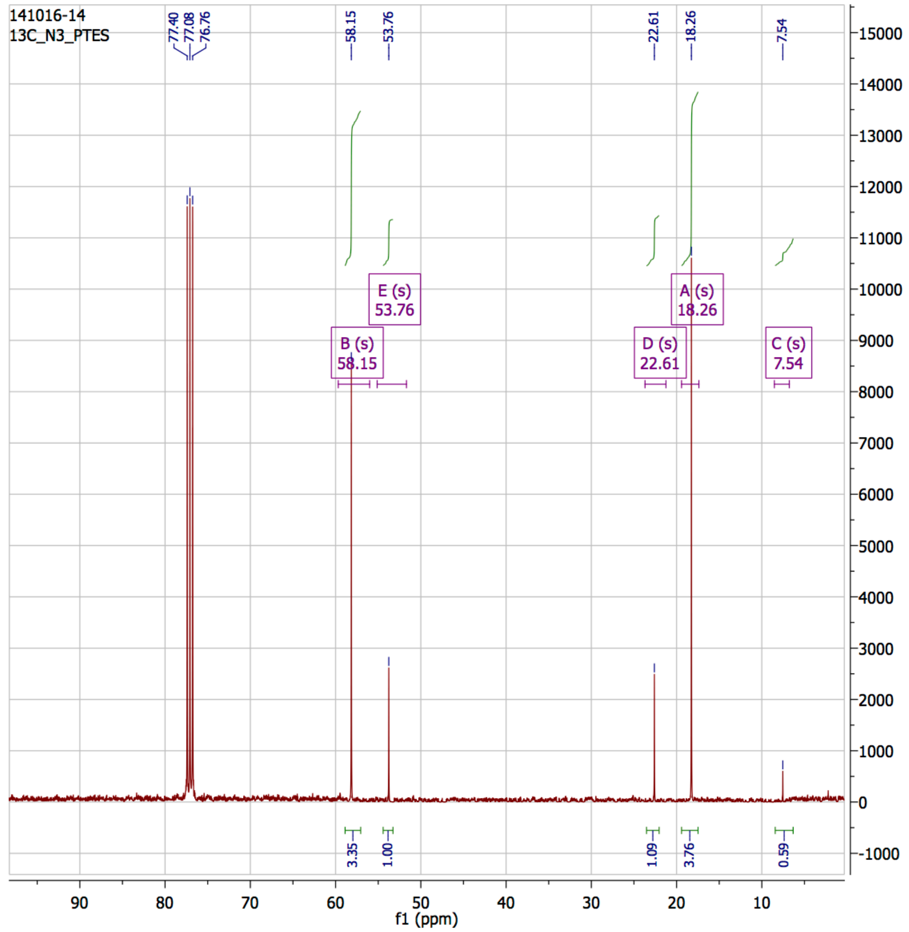

Supplementary Fig. 20 - NMR spectra of N_3PTES. (a) ^{1}H and (b) ^{13}C spectra. Full description of assigned peaks is provided in the Methods section.

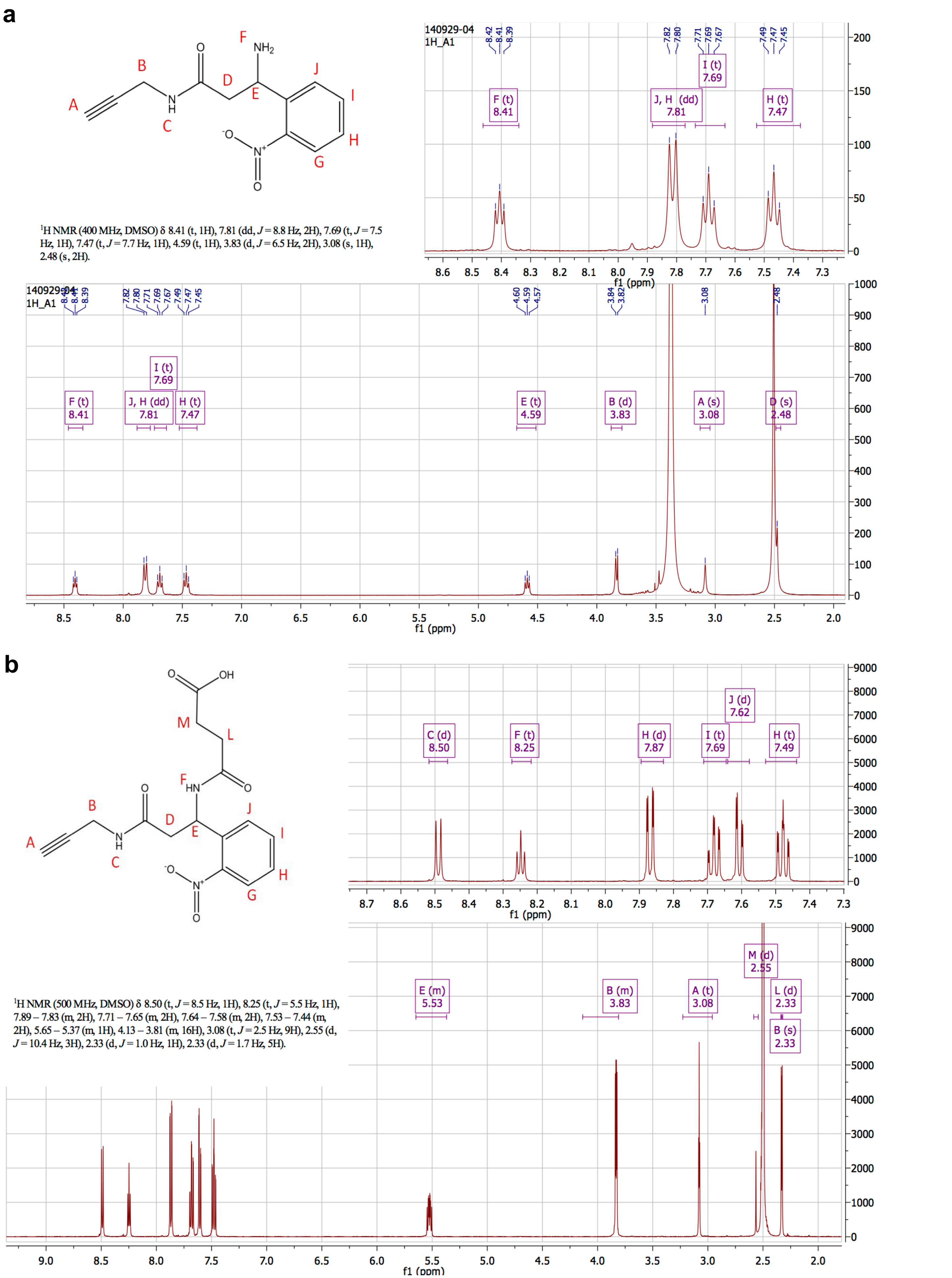

Supplementary Fig. 21 - ^{1}H NMR spectra of PCL. Synthesis of PCL was monitored by ^{1}H NMR of (a) compound 1 and (b) compound 2. Full description of assigned peaks is provided in the Methods section.

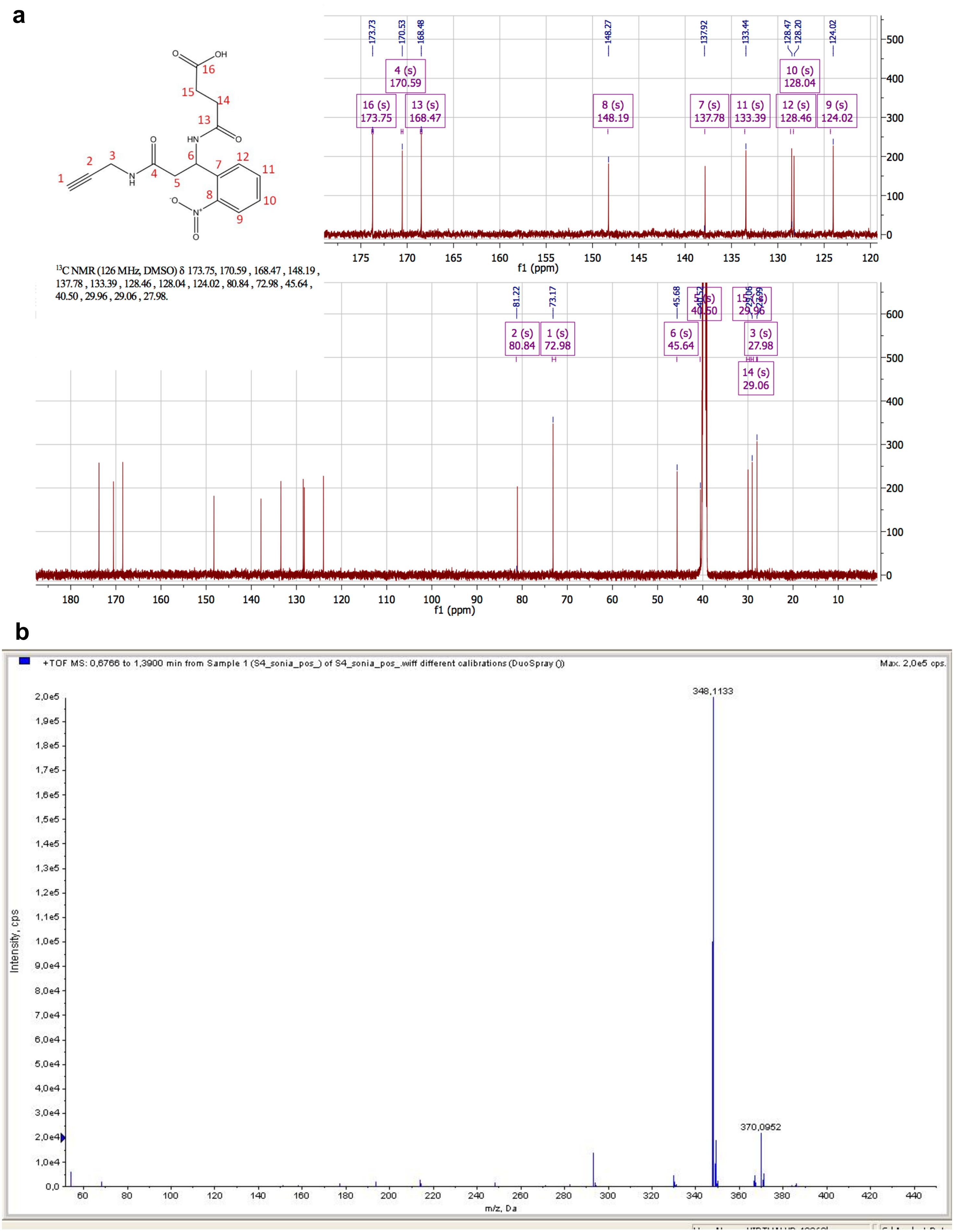

Supplementary Fig. 22 - Characterization of PCL. (a) ^{13}C NMR and (b) mass spectra (ESI Q-TOF) of PCL. Full description of assigned peaks is provided in the Methods section.

SUPPLEMENTARY METHODS

Production of recombinant nlsCre. Recombinant nlsCre protein was purchased from X-PROT (Cantanhede, Portugal). Briefly, an in-house construct containing the nlsCre in frame with a GST tag (glutathione s-transferase) was cloned in a pGEX family vector for transfection in *E. coli* BL21-codonplus strain (Agilent). Protein expression was induced with 0.5 mM isopropyl β-D-1-thiogalactopyranoside and the process was carried out overnight at 28 ºC. After expression, bacterial cultures were centrifuged at 3000 g, for 15 min at 4 ºC and the sediment was resuspended in PBS supplement with 0.5% Triton X-100. Cells were mechanically lysed using an Emulsiflex device and the protein soluble fraction was recovered after a centrifugation step at 3000 g, for 20 min at 4 ºC. The recombinant GST-nlsCre was then captured by affinity chromatography using glutathione s-transferase 4B resin (GE healthcare) and the recombinant nlsCre was obtained after incubation with 10 U/mL thrombin (GE healthcare) for 16 h at 4 ºC with gentle mixing. The enzymatic digestion was stopped by adding to the mixture 0.5 M Na_2HPO_4 (1/5 volume) followed by an incubation for 3 h at room temperature. In order to improve protein purity, the eluate was diluted 5× in 20 mM HEPES buffer pH 7.7 and then loaded to a cationic exchange column Mono-S (GE healthcare). The purified nlsCre was eluted with a linear gradient of NaCl (0-1 M) in 20 mM HEPES pH 7.7 buffer at a 1 mL/min flow. Protein quantification was determined by absorbance reading at 280 nm using a NanoDrop apparatus and protein purity was estimated by SDS-PAGE with Coomassie staining.

Labelling of Cre–UCNPs. Dylight 488-Cre-UCNPs were obtained by reacting the Cre- UCNPs with Dylight 488 NHS ester (ThermoFisher). In brief, Cre- UCNPs (1.5 mg) were dispersed in PBS (200 µL) and allowed to react with Dylight 488 NHS ester (1.6 µg) for 1 h. NPs were washed in water *via* centrifugation (6000 rpm, 3824 g, 6 min) and re-suspended in 100 µL of Tris-buffer at a final concentration of 12 mg/mL.

Preparation of UCNPs: synthesis of 3-(azidopropyltriethoxysilane N_3PTES). To a round bottom flask (100 mL), 3-chloropropyltriethoxysilane (3.11 mL, 16.6 mmol), sodium azide (2.16 g, 33.2 mmol), and butanone (50 mL) were added, under nitrogen atmosphere. The reaction mixture was heated under reflux for 72 h. After cooling down, the mixture was filtered over celite and the solvent was evaporated under vacuum. The crude mixture was then diluted with diethyl ether and filtered under inert atmosphere. The diethyl ether was removed in vacuum and the crude oil obtained was distilled under reduced pressure to yield a syrup as product (3.91 g, 96%) that was directly used without further purification. ^{1}H NMR (400 MHz, $CHCl_3$-d): δ 3.79 (q, J = 6.9 Hz, 6H), 3.24 (t, J = 6.4 Hz, 2H), 1.74 – 1.60 (m, 2H), 1.26 – 1.12 (m, 9H), 0.64 (dt, J = 11.7, 6.3 Hz, 2H). ^{13}C NMR (400 MHz, $CHCl_3$-d): δ 58.15, 53.76, 22.61, 18.26, 7.54. ^{1}H and ^{13}C NMR spectra were acquired using a Bruker Avance III 400 MHz spectrometer (Supplementary Figure 20). Chemical shifts (δ, ppm) were reported using TMS as an internal reference.

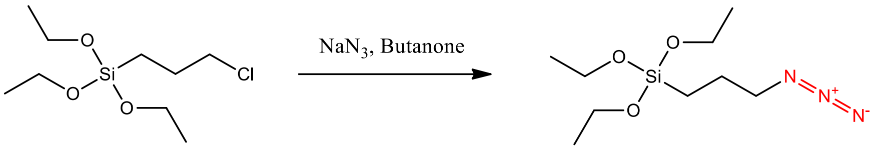

Supplementary Fig. 23. Schematic representation of the reaction between 3-chloropropyltriethoxysilane and sodium azide.

Preparation of UCNPs: synthesis and characterization of PCL. Synthesis of compound 1: 3-(9-fluorenylmethyloxycarbonyl)amino-3-(2-nitrophenyl)propionic acid (199.5 mg, 0.461 mmol, Iris biotech), 1-hydroxybenxzotriazole (HOBt, 74.8 mg, 0.554 mmol, Fluka) and (2-(1H-benzotriazol-1-yl)-1,1,3,3-tetramethyluronium hexafluorophosphate) (HBTu, 210 mg, 0.554 mmol) were dissolved in dichloromethane (7 mL). Subsequently, propargylamine (32.5 µL, 0.51 mmol Aldrich) dissolved in N,N-diisopropylethylamine (DIPEA 200 µL, 1.16 mmol) was added dropwise. The reaction mixture was allowed to react at room temperature for 90 min, light protected. A precipitate was formed and filtered, dried with Na_2SO_4 and analyzed by TLC showing a ≥ 99.9% purity. The yield

was 79% (176.8 mg). The compound obtained was used without any further purification in the following step. A volume of 400 µL of piperidine (4.08 mmol, Aldrich) was added to a suspension of the previous compound (170.0 mg, 0.36 mmol) in 4.0 mL of dimethylformamide (DMF) at room temperature and in the dark. The mixture was then stirred at room temperature for 2 h, the resulting crude concentrated under reduced pressure to eliminate the excess of piperidine and finally purified by silica column chromatography (CH_2Cl_2:MeOH, ramped 0 → 20% MeOH). Organic fractions were analyzed by TLC where the desired product was then collected and solvent was eliminated under reduced pressure. A yellow oil (89 mg, 94% yield) was identified as the desired product. ^{1}H-NMR (400 MHz, DMSO-d^6) δ: 8.51 (d, 1H), 8.41 (t, 2H), 7.81 (dd, 2H), 7.69 (t, 1H), 7.47 (t, 1H), 4.59 (t, 1H), 3.83 (d, 2H), 3.08 (s, 1H), 2.48 (s, 2H) (Supplementary Figure 21a). ^{13}C-NMR (101 MHz, DMSO-d^6) δ: 169.84, 148.62, 140.03, 132.99, 128.58, 127.86, 123.61, 81.10, 73.03, 47.47, 43.58, 27.78.

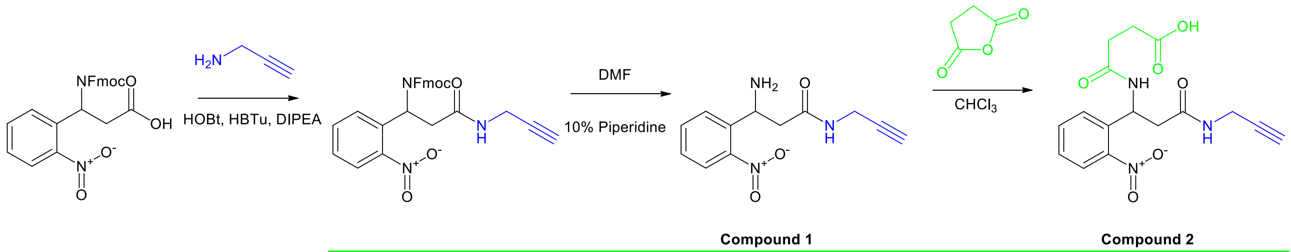

Supplementary Fig. 24. Schematic representation of the synthesis of photocleavable linker (PCL, compound 2). This compound was generated by a ring-opening amidation reaction of succinic anhydride with compound 1, which was first synthesized following the conjugation of the carboxylic acid with propargylamine.

Synthesis of compound 2 (4-((1-(2-nitrophenyl)-3-oxo-3-(prop-2-yn-1-ylamino)propyl)amino)-4-oxobutanoic acid): a solution of compound 1 (60.0 mg, 0.24 mmol) in 1.0 mL of $CHCl_3$ was added to a solution of succinic anhydride (29.5 mg, 0.27 mmol, Aldrich) also in 1.0 mL of $CHCl_3$ at room temperature. The temperature of the resulting mixture was increased to 70 ºC and stirred in the dark for 4 h. The resulting precipitate was then filtered and a white solid was obtained after drying under reduced pressure. The yield was 87% (73.3 mg). ^{1}H NMR (500 MHz, DMSO-d) δ 8.50 (d, 1H), 8.25 (t, 1H), 7.89 – 7.83 (d, 1H), 7.71 – 7.65 (t, 1H), 7.64 – 7.58 (d, 1H), 7.53 – 7.44 (t, 1H), 5.65 – 5.37

(m, 1H), 3.83 (m, 2H), 3.08 (t, 1H), 2.55 (d, 2H), 2.33 (m, 4H) (Supplementary Figure 21b). ^{13}C NMR (126 MHz, DMSO-d) δ 173.75, 170.59, 168.47, 148.19, 137.78, 133.39, 128.46, 128.04, 124.02, 80.84, 72.98, 45.64, 40.50, 29.96, 29.06, 27.98 (Supplementary Figure 22a). HRMS (ESI Q-TOF): $[MH]^+$ calcd. for $C_{16}H_{18}N_3O_6$ = 348.12, found: m/z 348.11 (Supplementary Figure 22b).

Characterization of the NPs: size and charge. The diameter of UCNPs before and after silanization was determined by TEM images were recorded on a Hitachi H9000-NA transmission electron microscope (TEM). Diluted UCNP suspensions (in H_2O) were placed on a 400-mesh 3 mm copper grid coated with a carbon support film (Taab Labs Ltd.) and dried overnight. The zeta-potential and NP counts of the NPs were measured by photon correlation spectroscopy (PCS) using quasi-elastic light scattering equipment (Zeta-PalsTM Zeta Potential Analyzer, Brookhaven Instruments Corp.), and ZetaPlusTM Particle Sizing Software (version 4.03). The zeta potential of NPs was determined in a 1 mM KCl pH 7 solution, DMEM and DMEM+10% FBS at 25°C. All data were recorded with at least 5 runs in triplicate.

Characterization of the NPs: composition. The powder X-ray diffraction (XRD) patterns of the dried UCNPs powder samples were collected on a Philips X'Pert MPD Powder X-ray diffractometer (CuK_α radiation at 1.54 Å) in the 2θ range 5 - 50°, with a 0.04° step scan mode. The Fourier transform infrared spectroscopy (FTIR) spectra were recorded on a Bruker IFS Equinox 55FTIR spectrometer (signal averaging 64 scans at a resolution of 4 cm^{-1} in KBr pellets containing ca. 2 mass % of material). Absorbance spectra were recorded on a Synergy H1 Multi-Mode Reader from Biotek.

Characterization of the NPs: photoluminescence properties. The upconversion emission spectra of suspensions of UCNPs and N_3/NH_2-UCNPs were recorded at 300 K using a Fluorolog®-3 Horiba Scientific (Model FL3-2T) spectroscope, with a TRIAX 320 single-emission monochromator (fitted with a 1200 grooves/mm grating blazed at 500 nm, reciprocal linear density of 2.6 $nm.mm^{-1}$), coupled to a R928 Hamamatsu photomultiplier. The excitation source was a 980 nm continuous wave (CW)

laser diode (Thorlabs LDC220 Laser diode controller 2A and TED200 Temperature controller) for up-conversion spectra measurements. A long pass glass filter (Thorlabs FGL780S) was used to cut the half-order harmonic of the laser. The emission spectra were corrected for detection and optical spectral response of the spectrofluorimeter.

Characterization of the NPs: amine quantification. The quantification of amine groups on the surface of the UCNPs was determined by measuring the fluorescence of fluorophore obtained from the reaction with fluorescamine (4-phenylspiro-[furan-2(3H),1-phthalan]-3,3′-dione) and the primary amines. A series of standard dilutions of glycine (Sigma) ranging from 0 to 500 μM were made using phosphate buffered saline (PBS) pH 7.4 as the diluent. After dilution, aliquots of samples (175 μL, containing 0.4 mg of N_3/NH_2-UCNPs) and standards (for the calibration curve and 0.4 mg of non-modified UCNPs) were pipetted into microplate wells in replicates of three. The 96 well black microplates with clear bottoms was placed on a microplate shaker and fluorescamine (25 μL of 21.6 mM (6 mg/mL), Sigma) dissolved in acetone was added to each well. Following the addition of fluorescamine the plate was shaken for one minute. The fluorescence was then determined using a Synergy H1 Multi-Mode Reader from Biotek with a 400 nm excitation filter (20 nm bandwidth) and a 460 nm emission filter (20 nm bandwidth). The sensitivity setting was at 70 and the data collected from the bottom with a 5 mm probe using static sampling with a 0.35 second delay, 50 reads per well.

Characterization of the NPs: HCQ quantification. Quantification of HCQ on the surface of the UCNPs was estimated by absorbance at 328 nm. A standard curve was obtained by simple dispersion of 1.5 mg of non-modified UCNPs with quantified amounts of HCQ in water ranging from 0 to 100 μM. The absorbance was determined on a Synergy H1 Multi-Mode Reader from Biotek. A quantification of 0.11 μM of HCQ was estimated for Cre-UCNPs (25 μg/mL).

Characterization of the NPs: release properties. Release of Cre from NPs was followed by irradiating samples at different times with a 980 nm laser at a power of 785 mW/cm^2. Briefly, Cre-UCNPs (164 µg) were photoactivated and the protein released from the system was collected in the supernatant after centrifugation (8000 rpm, 6797 g, 6 min). The collected fractions were denatured (95ºC, 5 min), loaded on a polyacrylamide gel (10%, w/v) and separated using Tris-Glycine-SDS running buffer (25 mM Tris, 192 mM Glycine and 0.1% w/v SDS). The gel was stained by a SimplyBlue^{TM} Safe Stain (ThermoFisher Scientific) according to the manufacturer's instructions. Cre protein was used to create a calibration curve: 0 µg; 0.3 µg; 0.6 µg; 1.25 µg and 2.5 µg. To quantify the overall concentration of Cre immobilized in UCNPs, the formulation was irradiated with a UV light for 20 min.

NP-cell interactions: cytotoxicity. For the cell viability assay, Cre reporter fibroblasts (1×10^4 cells/well) were seeded onto a 96-well plate and left to adhere. The cells were then incubated for 4 h with Cre-UCNPs (25, 50, 100, 250, 500 µg/mL), washed three times with cell medium to remove non-internalized NPs and cultured for additional 20 or 44 h. At these time points, ATP production was measured by a Celltiter-Glo Luminescent Cell Viability Assay (Promega) and cell number by a Neubauer chamber. For the annexin V/propidium iodide (PI) assay, fibroblasts (4×10^4 cells/well) were seeded on a 24 well plate and left to adhere overnight. The cells were then incubated with Cre-UCNPs (25, 50, 100, 250, 500 µg/mL) for 4 h, washed three times with warm PBS to remove non-internalized NPs, and incubated for additional 20 or 44 h. Then, the medium with the detached cells was collected and the adherent cells rinsed with PBS and trypsinized. Both the detached cells and adherent cells were then mixed and the cells were centrifuged for 3 min at 300 g. The cells were washed with PBS, resuspended in PBS (100 µL), and stained with annexin binding buffer (Invitrogen, 200 µL, containing 2.5 µL of annexin V-FITC conjugate). The cells were incubated in the dark at

room temperature for 15 min and then on ice before analysis in the flow cytometer. To stain the dead cells, PI (100 μL, 3 μg/mL) was added shortly before running each sample.

NP-cell interactions: heat-shock proteins. Cre reporter fibroblasts (4×10^4 cells/well) were incubated with Cre-UCNPs (50 μg/mL) for 4 h in serum-free DMEM medium and washed with warm PBS. Cells were irradiated for 5, 10, 15 or 30 min with NIR-light (785 mW/cm^2, 980 nm). As a positive control, cells were incubated at 43ºC for 1 h. As negative controls, cells were incubated without Cre-UCNPs for 4 h and then irradiated (5, 10, 15 or 30 min) or incubated with Cre-UCNPs for 4 h and non-irradiated. Immediately after irradiation cells were fixed with 4% (v/v) paraformaldehyde and incubated with mouse anti-human HSP70 (3A3) antibody (1:50, sc-32239, Santa Cruz) for 1 h at room temperature. As a secondary antibody, cells were then incubated for 1 h in the dark with a secondary antibody sheep IgG-Cy3 anti-mouse (1:100, Sigma). Cell nuclei were stained with DAPI (Sigma) and the slides were mounted with mounting medium (Dako) and examined with a Zeiss LSM 710 confocal microscope using a Apochromat 40x/1.4 objective. Nuclear localization of HSP70 was quantified by colocalization between the nuclear staining (DAPI) and the HSP70 staining. This analysis was performed with the JACop plug-in for ImageJ through the Mander's overlap coefficient.

NP-cell interactions: H2A.X phosphorylation. Cre reported fibroblasts (4×10^4 cells/well) were incubated with Cre-UCNPs (50 μg/mL) for 4 h in serum-free medium, after which the medium was changed and cells were irradiated for 5, 10, 15 or 30 min with NIR-light (785 mW/cm^2, 980 nm). As a positive control, cells were irradiated for 30 min with near-UV pulsed light (405 nm, 94 mW/cm^2). As negative control, cells were cultured in the absence of irradiation. At the end of all the procedures, the cells were fixed with 4% (v/v) paraformaldehyde, permeabilized with triton-X (0.3%, w/v) and blocked with 1% (w/v) BSA and 0.3 M glycine. Cells were then incubated with mouse anti γ-H2A.X

(phosphor S139) antibody (ab11174, Abcam) (1:1000 in PBS containing 1% BSA) for 1 h at room temperature. As a secondary antibody, cells were incubated for 1 h in the dark with goat anti-rabbit IgG Alexa 488 antibody (1:100 dilution in PBS containing 1% BSA). Cell nuclei were stained with DAPI (Sigma), and the slides were mounted with mounting medium (Dako) and examined with a Zeiss LSM 710 confocal microscope using an oil immersion Apochromat 40x/1.4 objective.

NP cellular uptake and intracellular trafficking by TEM analyses. Cells were fixed by immersion in 2.5% glutaraldehyde and 2% paraformaldehyde in 0.1 M sodium cacodylate buffer pH 7.4 solution for 5 days. After washing and 2 h in post-fixating 2% osmium tetroxide in 0.1 M sodium cacodylate buffer pH 7.4 solution, tissues were washed in buffer, incubated with 1% uranyl acetate overnight, washed in buffer and dehydrated through graded series of ethanol, and embedded in Epon (EMS). Ultrathin sections were cut at 50 nm and prepared on a RMC Ultramicrotome (PowerTome, USA) using a diamond knife and recovered to 200 mesh Formvar Ni-grids, followed by 2% uranyl acetate and saturated lead citrate solution. Visualization was performed at 80 kV in a (JEOL JEM 1400 microscope (Japan)) and digital images were acquired using a CCD digital camera Orious 1100 W (Tokyo, Japan). The transmission electronic microscopy was performed at the HEMS core facility at i3S, University of Porto, Portugal.

***In vitro* Cre-mediated DNA recombination using a commercial transfection agent.** Cre reporter fibroblasts (4×10^4) were seeded onto a 24-well plate and left to adhere overnight. Cells were incubated with different concentrations of Cre-UCNPs (1.5 - 50 μg/mL) and Cre-UCNPs without HCQ (1.5 - 50 μg/mL) for 4 h, washed with PBS, and left to grow in complete medium for 48 h. In a separate experiment, cells were incubated with nls-Cre recombinase (1.9 - 750 nM) complexed with lipofectamine RNAiMAX (1.5 μL, Life Technologies) in antibiotic-free complete medium for 24 h. After 24 h, the media was replaced with fresh full serum media. All complexing steps were performed

at room temperature. Non-transfected cells were used as a negative control. For the assessment of positive cells percentage, flow cytometry was used. For this purpose, cells were washed with cold trypan blue solution, re-washed three times with cold PBS, dissociated with trypsin (0.1%, w/v, in PBS), centrifuged and ressuspended in PBS for flow cytometry analyses.

***In vitro* deep-tissue activation.** Cre reporter fibroblasts (5×10^3) were seeded onto a 96-well plate and left to adhere overnight. The cells were incubated with Cre-UCNPs (50 µg/mL) for 4 h, washed to remove the non-internalized NPs, and irradiated with a NIR laser (980 nm, 3 cycles of 5 min irradiation, at a power of 425 mW/cm^2). Photoactivation was performed through mice tissues of different thickness and composition: skin (0.5 mm), ribs (3 mm), whole brain (6 mm). Efficiency of laser penetration was evaluated overtime by direct analysis of GFP expression (mean green intensity in the cytoplasm of Cre reporter cells) in cells by the IN Cell Analyzer 2200 cell imaging system, using an inverted 20x PlanFluor objective/0.45 numerical aperture. Cell recombination was detected using an excitation filter 475/28 and an emission filter 511.5/23.

Animal testing: isolation of SVZ cells. SVZ cell cultures were prepared from 1 to 3 days-old R26YFP mice. For this purpose, brains were removed from the skull and placed in Hank's balanced salt solution (HBSS) containing penicillin (100 U/mL) and streptomycin (100 µg/mL, Life Technologies). Coronal brain sections (450 µm thick) where obtained using a McIlwain tissue chopper and SVZ fragments were subsequently dissected. SVZ was digested in trypsin (0.025%, w/v, Life Technologies) and EDTA (0.265 mM, Life Technologies), followed by mechanical dissociation. The resulting single cell suspension was grown in serum-free medium (SFM) composed of Dulbecco's modified Eagle medium [(DMEM)/F12 + GlutaMAX™-l)] supplemented with B27 supplement (1%), epidermal growth factor (10 ng/mL, EGF), basic fibroblast growth factor-2 (5 ng/mL, FGF-2) and penicillin (100 U/mL), streptomycin (100 µg/mL; all from Life Technologies) on

uncoated petri dishes in an incubator with 5% CO_2 and 95% atmospheric air at 37 °C. Five to six-days after, these SVZ cells grown in suspension and generated neurospheres. At these time-point, neurospheres were dissociated using the commercial NeuroCult™ Chemical Dissociation Kit (StemCell Technologies) according to manufacturer's instructions and the resulting single cells were seeded onto 24-well plate poly-d-lysine-coated glass coverslips at 10^5 cells per well for all the remaining experiments in SFM medium supplemented with EGF (2.5 ng/mL) and FGF-2 (1.25 ng/mL). Two days after, cells were incubated with Cre-UCNPs for 4 h, followed by the activation of NIR laser (980 nm; 425 mW/cm^2; 3 cycles of 5 min irradiation). As positive control, SVZ cells were infected with AAV5.Cre (Supplementary Table 1; 1 µL/mL). Values of Cre-UCNPs-mediated recombination were compared with soluble nls-Cre and HCQ at the same concentration that was present in the NPs (i.e. 200 µg/mL=1.9 µg/mL Cre + 0.88 µM HCQ). After 4 days, the cells were fixed with 4% (v/v) paraformaldehyde, permeabilized and blocked for 1 h with 6% (w/v) BSA and 0.25% (w/v) Triton X-100 in PBS. Cells were then incubated with goat anti-GFAP antibody (ab53554, Abcam) (1:500), mouse anti-Nestin antibody (ab6142, Abcam) (1:1000), mouse anti-NeuN antibody (MAB377, Milipore) (1:50) and goat anti-DCX (Santa Cruz) (1:200) in PBS overnight at 4ºC. As a secondary antibody, cells were incubated for 1 h in the dark with donkey anti-mouse IgG Alexa 594 antibody, donkey anti-goat IgG Alexa 647 antibody and donkey anti-rabbit Alexa 488 (1:1000 dilution in PBS). Cell nuclei were stained with DAPI (Sigma), and the slides were mounted with fluorescent mounting medium (Dako) and examined with a Zeiss LSM 710 confocal microscope using an oil immersion Apochromat 40x/1.4 objective.

Animal testing: intracellular accumulation of Cre-UCNPs in SVZ cells by TEM analyses. Tissues were perfused with PBS and fixed by immersion in 2.5% glutaraldehyde and 2% paraformaldehyde in 0.1 M sodium cacodylate buffer pH 7.4 solution for 5 days. After washing and 2 h in post-fixating 2% osmium tetroxide in 0.1 M sodium cacodylate buffer pH 7.4 solution, tissues

were washed in buffer, incubated with 1% uranyl acetate overnight, washed in buffer and dehydrated through graded series of ethanol, and embedded in Epon (EMS). Ultrathin sections were cut at 50 nm and prepared on a RMC Ultramicrotome (PowerTome, USA) using a diamond knife and recovered to 200 mesh Formvar Ni-grids, followed by 2% uranyl acetate and saturated lead citrate solution. Visualization was performed at 80 kV in a (JEOL JEM 1400 microscope (Japan)) and digital images were acquired using a CCD digital camera Orious 1100 W (Tokyo, Japan). The transmission electronic microscopy was performed at the HEMS core facility at i3S, University of Porto, Portugal.

Preparation of neuronal cultures. Cortices of E17-E19 Wistar rat embryos were dissected and treated with 0.06% trypsin for 15 min at 37ºC. Following dissection in Ca^{2+} and Mg^{2+}-free Hank's balanced salt solution (HBSS) composed of (in mM) 5.36 KCl, 0.44 KH_2PO_4, 137 NaCl, 4.16 $NaHCO_3$, 0.34 $Na_2HPO_4.2H_2O$, 5 glucose, 1 sodium pyruvate, 10 HEPES and 0.001% phenol red, ~4.7×10^4 cells per/cm^2 were seeded in 12-well multi-well plates containing coverslips previously coated with poly-D-lysine (0.1 mg/mL), in neuronal plating medium (MEM supplemented with 10% horse serum, 0.6% glucose and 1 mM pyruvic acid). After 2-4 h, media was replaced for neuronal culture medium (NBM supplemented with SM1 supplement [1:50 dilution], 0.5 mM glutamine, 0.12 mg/mL gentamycin). Cells were maintained at 37ºC with 5% CO_2 and 1/3 of the neuronal culture medium was replaced once before infection. After 7 days in culture, cells were infected with AAV5.DIO.ChR2.YFP (Supplementary Table 1). Briefly, 300 µL of the medium of the well was removed (conditioned medium) and the same amount of fresh medium containing 2.95×10^9 GC/mL. After 3 days, cells were exposed to Cre-UCNPs (100 µg/mL) for 4 h, washed, activated by NIR laser (980 nm; 785 mW/cm^2; 3 cycles of 5 min irradiation) and allowed to recover in the previous conditioned medium for another 3 days. Cells were then analyzed for ChR2-YFP expression by flow cytometry in a BD Accuri™ C6 flow cytometer or processed for electrophysiology and optogenetics studies. In the flow cytometry studies, percentage of positive cells was calculated based in the basal

expression of AAV9.ChR2-YFP (Supplementary Table 1) in non-treated cortical neurons (1% overlap).

***In vitro* electrophysiology and optogenetics.** Whole-cell patch clamp recordings were performed in the *in vitro* cultures of cortical neurons at days 9-11, at room temperature (25° C). For all experiments, the extracellular solution was composed of (in mM): 140 NaCl, 2.4 KCl, 10 HEPES, 10 Glucose, 2 $MgCl_2$ and 1 $CaCl_2$. Visualization of cortical neurons was achieved with a Axio Examiner.D1 microscope (Zeiss) equipped with a 40× water immersion objective and a Q-capture Pro7 camera (QImaging) and infected cells were identified by high GFP expression. Borosilicate glass (Science Products) were used as recording electrodes (3-5 MΩ) filled with an intracellular solution containing (in mM): 110 cesium methanesulfonate, 30 tetraethylammonium chloride, 10 EGTA, 2 $MgCl_2$, 0.1 $CaCl_2$ and 10 mM HEPES (pH 7.25, 290 mOsm). Both ChR2- and infrared-mediated currents were elicited by placing an optic fiber attached to a LED module (Plexon) in the left side of the microscope optic field. To elicit ChR2 currents, a blue light (465 nm LED module; 40 mA ≈ 3.4 mW/cm^2; 90 mA ≈ 4.2 mW/cm$^{2;}$ 300 mA ≈ 7.2 mW/cm^2) was used, while in the IR-mediated currents were induced with a 980 nm wavelength. In all cases, neurons were clamped at -70 mV and currents were measured in voltage-clamp mode. Criteria for acceptance of cells was determined as a stable Ra under 20 MΩ. Recordings were filtered at 2 kHz and digitized at 20 kHz. Data was acquired with a Multiclamp 700B amplifier and Digidata 1550A (Molecular Devices Corporation) and analyzed using Clampfit 10.7 software (Axon Instruments).

Animal testing: intramuscular transplantation of Cre-UCNPs-treated cells. Prior to transplantation, Cre reporter fibroblasts were plated on 24 well plates and treated with treated with Cre-UCNP (50 µg/mL). After 4 h, the cells were washed three times with PBS, dissociated with trypsin (0.1%, w/v, in PBS), centrifuged and counted. Cells were then injected in gastrocnemius

(limb) muscles of a male C57/BL6 mouse (8 weeks old), activated by a NIR laser at 980 nm (425 mW/cm^2; 3 cycles of 5 min irradiation) directly above the injection site. Recombination was evaluated 60 h after injection. As a positive control, a group of animals were injected with cells subjected to the same NIR activation protocol prior to injection (*in vitro*). As a negative control, a group of animals were injected with non-irradiated Cre reporter fibroblasts. To monitor cell recombination, animals were sacrificed and perfused with a 10% solution of neutral buffered formalin (HT501320, Sigma). Gastrocnemius muscles were harvested and further fixed overnight in the same fixative. Before freezing, tissues were cryo-protected in 30% sucrose (w/v) in PBS. Gastrocnemius muscles from the hind limb of mice were imbedded in OCT compound, snap-frozen and sectioned on a Cryostast (Leica) into 10 µm thick sections. Sections were then washed three times in PBS, stained with the DAPI (1 µg/mL) and mounted with Dako fluorescence mounting medium (Dako). Confocal fluorescent images were acquired using a laser scanning confocal microscope (LSM 710, Zeiss using an oil immersion Apochromat 40x/1.4 objective). Per injection, the hot two sections containing cell engraftments were chosen for image analysis and GFP-positive cells quantification. GFP intensity was measured using FIJI software and normalized to cell number (nuclei number).

Supplementary Table 1- List of viruses used.

Name	Reference	Titration (GC)
AAV5.DIO.ChR2.YFP	AAV5.EF1aDIO.hChR2 (H134R)-eYFP.WPRE.hGH, Penn Vector Core	1.77×10^{13}
AAV9.ChR2-YFP	AAV9.hSyn.hChR2(H134R)-eYFP.WPRE.hGH, Penn Vector Core	1.03×10^{14}
AAV5.Cre	AAV5.CMV.PI.Cre.rBG, Penn Vector Core	1.18×10^{12}

Supplementary Table 2- List of antibodies used.

Name /clone	Reference	Dilution	Secondary Antibody
mouse anti-HSP70 /3A3	sc-32239, Santa Cruz	1:50	*1
rabbit anti γ-H2A.X /polyclonal	ab11174, Abcam	1:1000	*2
mouse anti-Cre Recombinase /2D8	MAB3120, Merck Milllipore	1: 3000	*3 *10
rat anti-mouse galectin 9 /108A2	137901, BioLegend	1:100	*4
rabbit anti-EEA1 /C45B10	3288S, Cell Signaling	1:100	*5
rabbit anti-Rab 7 /D95F2	9367S, Cell Signaling	1:100	*5
rabbit anti-Lamp1 /polyclonal	ab24170, Abcam	1:1000	*2 *5
rabbit anti-GFP (YFP) /polyclonal	Ab6556, Abcam	1:1000	*5*6 *7
goat anti-GFAP /polyclonal	ab53554, Abcam	1:500	*8
mouse anti-GFAP /GA5	3670S, Cell signalling	1:200	*9
mouse anti-Nestin / Rat-401	ab6142, Abcam	1:1000	*9 *10
mouse anti-Sox2 /E-4	sc-365823, Santa Cruz	1:100	*9
mouse anti-NeuN antibody /A60	MAB377, Millipore	1:50	*9*10
goat anti-Doublecortin (DCX) /C-18	sc-8066, Santa Cruz	1:200	*8
mouse anti-Doublecortin (DCX) /E-6	sc-271390, Santa Cruz	1:50	*9
mouse anti-Channelrhodopsin 2 (ChR2) /15E2	610180, Progen	1:500	*9
mouse anti-Tyrosine Hydroxylase /LNC1	22941, ImmunoStar	1:5000	*9
rabbit anti-cFos /polyclonal	Ab7963, Abcam	1:500	*11
rabbit anti-Iba-1 /polyclonal	019-19741, Wako	1:1000	*7

Secondary antibodies:

*1 sheep anti-mouse IgG Cy3 (Sigma) 1:100
*2 goat anti-rabbit IgG Alexa 488 antibody (Life Technologies) 1:100
*3 goat anti-mouse IgG Alexa 488 (Life Technologies) 1:100
*4 rabbit anti-rat IgG Alexa 488 (Life Technologies) 1:1000
*5 goat anti-rabbit IgG Cy3 (Jackson ImmunoResearch) 1:100 /1:1000
*6 donkey anti-rabbit IgG Alexa 488 (Life Technologies) 1:200
*7 goat anti-rabbit IgG Alexa 633 (Life Technologies) 1:1000
*8 donkey anti-goat IgG Alexa 647 (Life Technologies) 1:200
*9 donkey anti-mouse IgG Alexa 633 (Life Technologies) 1:1000
*10 donkey anti-mouse IgG Alexa 594 (Life Technologies) 1:200
*11 donkey anti-rabbit IgG Alexa-568 (Life Technologies) 1:1000

Supplementary Table 3- List of primers used for RT-qPCR.

Gene	Accession no. (GenBank)	Primer	Sequence (5' → 3')
Cltc	NM_001003908.2	Forward	GCCCATTCGCTTTCAGGAGC
		Reverse	GATTACCACCTGGGCCTGCT
Ldlr	NM_010700.3	Forward	TGCCAATCGACTCACGGGTT
		Reverse	GAGTGGGGACCGATCTGTGG
Cav1	NM_007616.4	Forward	GCCCAGGGAAACCTCCTCAG
		Reverse	TGTGCGCGTCATACACTTGC
Cdc42	NM_009861.3	Forward	CCCGGCGGAGAAGCTGAG
		Reverse	CCAACAGCACCATCACCAAC
Rac1	NM_001347530.1	Forward	ACAGATTGCGTCCCCTCTCC
		Reverse	TGCCCCTGGAGGGTCTATCT
Ctbp1	NM_001198859.1	Forward	GAGCTCAATGGGGCTGCCTA
		Reverse	CAGGGACATGGCACTGGGAA
Scara3	NM_172604.3	Forward	ACCACGGAGAAATCCTTCGCA
		Reverse	TGCCAGGATCCCCTTTAGGTC
