# Supplementary material for: Efficient spatially targeted gene editing using a near-infrared activatable protein-conjugated nanoparticle for brain applications
Source: Nat Commun. 2022 Jul 16;13:4135. doi: 10.1038/s41467-022-31791-6 (PMC9287341; doi:10.1038/s41467-022-31791-6)
Supplement: Supplementary file 3 — Reporting Summary [file 41467_2022_31791_MOESM3_ESM.pdf]

nature portfolio

Corresponding author(s): Lino Ferreira
Last updated by author(s): Jun 9, 2022

Reporting Summary

Nature Portfolio wishes to improve the reproducibility of the work that we publish. This form provides structure for consistency and transparency in reporting. For further information on Nature Portfolio policies, see our Editorial Policies and the Editorial Policy Checklist.

Statistics

For all statistical analyses, confirm that the following items are present in the figure legend, table legend, main text, or Methods section.

n/a	Confirmed	
☐	☒	The exact sample size (*n*) for each experimental group/condition, given as a discrete number and unit of measurement
☐	☒	A statement on whether measurements were taken from distinct samples or whether the same sample was measured repeatedly
☐	☒	The statistical test(s) used AND whether they are one- or two-sided<br>*Only common tests should be described solely by name; describe more complex techniques in the Methods section.*
☒	☐	A description of all covariates tested
☒	☐	A description of any assumptions or corrections, such as tests of normality and adjustment for multiple comparisons
☐	☒	A full description of the statistical parameters including central tendency (e.g. means) or other basic estimates (e.g. regression coefficient) AND variation (e.g. standard deviation) or associated estimates of uncertainty (e.g. confidence intervals)
☐	☒	For null hypothesis testing, the test statistic (e.g. *F*, *t*, *r*) with confidence intervals, effect sizes, degrees of freedom and *P* value noted<br>*Give P values as exact values whenever suitable.*
☒	☐	For Bayesian analysis, information on the choice of priors and Markov chain Monte Carlo settings
☒	☐	For hierarchical and complex designs, identification of the appropriate level for tests and full reporting of outcomes
☒	☐	Estimates of effect sizes (e.g. Cohen's *d*, Pearson's *r*), indicating how they were calculated

Our web collection on statistics for biologists contains articles on many of the points above.

Software and code

Policy information about availability of computer code

Data collection	Data were collected using dedicated software to each equipment, as described in the Methods section. Behavioral data was acquired and processed using Bonsai, a free open-source visual programming environment (https://bitbucket.org/horizongir/bonsai).
Data analysis	All software used to analyze data is described in the Methods section. Image processing was performed using ImageJ/FIJI. Flow cytometry data was processed using FlowJo. Electrophysiological data was processed using Clampfit. Statistical analysis was performed using GraphPad Prism.

For manuscripts utilizing custom algorithms or software that are central to the research but not yet described in published literature, software must be made available to editors and reviewers. We strongly encourage code deposition in a community repository (e.g. GitHub). See the Nature Portfolio guidelines for submitting code & software for further information.

Data

Policy information about availability of data

All manuscripts must include a data availability statement. This statement should provide the following information, where applicable:
- Accession codes, unique identifiers, or web links for publicly available datasets
- A description of any restrictions on data availability
- For clinical datasets or third party data, please ensure that the statement adheres to our policy

All data generated or analyzed during this study are included in this article (and its Supplementary Information). All the figures have associated source data.

Field-specific reporting

Please select the one below that is the best fit for your research. If you are not sure, read the appropriate sections before making your selection.

- [x] Life sciences
- [ ] Behavioural & social sciences
- [ ] Ecological, evolutionary & environmental sciences

For a reference copy of the document with all sections, see nature.com/documents/nr-reporting-summary-flat.pdf

Life sciences study design

All studies must disclose on these points even when the disclosure is negative.

Sample size	No statistical methods were employed to predetermine sample sizes. Experimental sample sizes were chosen according to common practices in the field, which have also generated reproducible results (e.g. ref. 12 Morales et al., Small, 2018; ref. 16 Chen et al., Science, 2018; ref. 61 Lee et al., Nat Biomed Eng, 2018).
Data exclusions	No data were excluded from the analyses in this study.
Replication	The number of replicates used in all experiments is stated in the figure captions.
Randomization	No randomization was used to allocate animals to experimental groups.
Blinding	Samples were prepared unblinded and under same conditions between different treatment groups. Quantification of parameters was applied similarly across all groups and replicates. Blinding was applied during behavioral experiments.

Reporting for specific materials, systems and methods

We require information from authors about some types of materials, experimental systems and methods used in many studies. Here, indicate whether each material, system or method listed is relevant to your study. If you are not sure if a list item applies to your research, read the appropriate section before selecting a response.

Materials & experimental systems

n/a	Involved in the study	
☐	☒	Antibodies
☐	☒	Eukaryotic cell lines
☒	☐	Palaeontology and archaeology
☐	☒	Animals and other organisms
☒	☐	Human research participants
☒	☐	Clinical data
☒	☐	Dual use research of concern

Methods

n/a	Involved in the study	
☒	☐	ChIP-seq
☐	☒	Flow cytometry
☒	☐	MRI-based neuroimaging

Antibodies

Antibodies used

Primary
1. mouse anti-HSP70 (clone 3A3, sc-32239, Santa Cruz) = 1:50 dilution
2. rabbit anti γ-H2A.X (polyclonal, ab11174, Abcam) = 1:1000 dilution
3. mouse anti-Cre Recombinase (clone 2D8, MAB3120, Merck Milllipore) = 1:3000 dilution
4. rabbit anti-EEA1 (clone C45B10, 3288S, Cell Signaling) = 1:100 dilution
5. rabbit anti-Rab 7 (clone D95F2, 9367S, Cell Signaling) = 1:100 dilution
6. rabbit anti-Lamp1 (polyclonal, ab24170, Abcam) = 1:1000 dilution
7. rabbit anti-GFP (YFP) (polyclonal, ab6556, Abcam) = 1:1000 dilution
8. goat anti-GFAP (polyclonal, ab53554, Abcam) = 1:500 dilution
9. mouse anti-GFAP (clone GA5, 3670s, Cell signalling) = 1:200 dilution
10. mouse anti-Nestin (clone Rat-401, ab6142, Abcam) = 1:1000 dilution
11. mouse anti-Sox2 (clone E-4, sc-365823, Santa Cruz) = 1:100 dilution
12. mouse anti-NeuN antibody (clone A60, MAB377, Millipore) = 1:50 dilution
13. goat anti-Doublecortin (DCX) (clone C-18, sc-8066, Santa Cruz) = 1:200 dilution
14. mouse anti-Doublecortin (DCX) (clone E-6, sc-271390, Santa Cruz) = 1:50 dilution
15. mouse anti-Channelrhodopsin 2 (ChR2) (clone 15E2, 610180, Progen) = 1:500 dilution
16. mouse anti-Tyrosine Hydroxylase (clone LNC1, 22941, ImmunoStar) = 1:5000 dilution
17. rabbit Anti-cFos (polyclonal, ab7963, Abcam) = 1:500 dilution
18. rabbit Anti-IBA-1 (019-19741, Wako) = 1:1000 dilution
19. rat anti-galectin 9 (clone 108A2, 137901, BioLegend) = 1:100 dilution

Secondary
1. sheep anti-mouse IgG Cy3 (Sigma) = 1:100
2. goat anti-rabbit IgG Alexa Fluor 488 (Life Technologies) = 1:100
3. goat anti-mouse IgG Alexa Fluor 488 (Life Technologies) = 1:100
4. rabbit anti-rat IgG Alexa Fluor 488 (Life Technologies) = 1:1000
5. goat anti-rabbit IgG Cy3 (Jackson ImmunoResearch) = 1:100 /1:1000
6. donkey anti-rabbit IgG Alexa Fluor 488 (Life Technologies) 1:200
7. goat anti-rabbit IgG Alexa Fluor 633 (Life Technologies) 1:1000
8. donkey anti-goat IgG Alexa Fluor 647 (Life Technologies) 1:200
9. donkey anti-mouse IgG Alexa Fluor 633 (Life Technologies) 1:1000
10. donkey anti-mouse IgG Alexa Fluor 594 (Life Technologies) 1:200
11. donkey anti-rabbit IgG Alexa Fluor 568 (Life Technologies) 1:1000

Validation

All antibodies are commercially available and were validated for ICC/IHC by their manufacturers.

Eukaryotic cell lines

Policy information about cell lines

Cell line source(s)	Cell line: SFr-II (derived from SC-1 mouse fetal embryo fibroblasts)<br>Source: Dr Carol Stocking, University of Hamburg, Germany (https://doi.org/10.1093/nar/gnf059)
Authentication	None of the cell lines used were authenticated.
Mycoplasma contamination	Cells were confirmed to be negative for mycoplasma contamination using the MycoAlert Detection Kit (Lonza).
Commonly misidentified lines<br>(See ICLAC register)	No commonly misidentified cell lines were used.

Animals and other organisms

Policy information about studies involving animals; ARRIVE guidelines recommended for reporting animal research

Laboratory animals	Strain: R26tdTomato (in C57BL/6J background)<br>Source: Jackson<br><br>Strain: R26-YFP (in C57BL/6J background)<br>Source: Dr Henrique Veiga-Fernandes (IMM, Lisbon, Portugal)<br><br>Strain: C57/BL6 (wildtype)<br>Source: Charles River
Wild animals	This study did not involve wild animals.
Field-collected samples	This study did not involve sample collection in the field.
Ethics oversight	All experiments were carried with the approval of the animal ethics committee of the Center for Neuroscience and Cell Biology, University of Coimbra (ORBEA), the approval of the Portuguese DGAV, and in accordance with EU directives regarding animal use in research.

Note that full information on the approval of the study protocol must also be provided in the manuscript.

Flow Cytometry

Plots

Confirm that:

- [x] The axis labels state the marker and fluorochrome used (e.g. CD4-FITC).
- [x] The axis scales are clearly visible. Include numbers along axes only for bottom left plot of group (a 'group' is an analysis of identical markers).
- [ ] All plots are contour plots with outliers or pseudocolor plots.
- [x] A numerical value for number of cells or percentage (with statistics) is provided.

Methodology

Sample preparation

Cells were trypsinised and washed with PBS before staining and analysis in the cytometer. Each experiment is described in detail in the Methods section.

Instrument	BD Accuri C6
Software	FlowJo (version X.0.7)
Cell population abundance	Cells were abundant, with >80% of the events passing the "singlets" gate.
Gating strategy	Gating strategy is described in Supplementary Figure 15. After FSC/SSC gating followed by FSC-H vs FSC-A, GFP+ and YFP+ cell populations were selected from singlets based on negative controls.

☒ Tick this box to confirm that a figure exemplifying the gating strategy is provided in the Supplementary Information.
